# Transcriptomic Changes in *Medicago truncatula* and *Lotus japonicus* Root Nodules during Drought Stress

**DOI:** 10.3390/ijms20051204

**Published:** 2019-03-09

**Authors:** Izabela Sańko-Sawczenko, Barbara Łotocka, Jakub Mielecki, Hanna Rekosz-Burlaga, Weronika Czarnocka

**Affiliations:** 1 Department of Botany, Faculty of Agriculture and Biology, Warsaw University of Life Sciences, Nowoursynowska 159, 02-776 Warsaw, Poland; izabela_sanko_sawczenko@sggw.pl (I.S.-S.); barbara_lotocka@sggw.pl (B.Ł.); 2 Department of Plant Genetics, Breeding and Biotechnology, Faculty of Horticulture, Biotechnology and Landscape Architecture, Warsaw University of Life Sciences, Nowoursynowska 159, 02-776 Warsaw, Poland; jakub_mielecki@sggw.pl; 3 Department of Microbial Biology, Faculty of Agriculture and Biology, Warsaw University of Life Sciences, Nowoursynowska 159, 02-776 Warsaw, Poland; hanna_rekosz_burlaga@sggw.pl

**Keywords:** Fabaceae, drought, abiotic stress, nodules, rhizobia, RNA-Seq

## Abstract

Drought is one of the major environmental factors limiting biomass and seed yield production in agriculture. In this research, we focused on plants from the Fabaceae family, which has a unique ability for the establishment of symbiosis with nitrogen-fixing bacteria, and are relatively susceptible to water limitation. We have presented the changes in nitrogenase activity and global gene expression occurring in *Medicago* *truncatula* and *Lotus* *japonicus* root nodules during water deficit. Our results proved a decrease in the efficiency of nitrogen fixation, as well as extensive changes in plant and bacterial transcriptomes, shortly after watering cessation. We showed for the first time that not only symbiotic plant components but also *Sinorhizobium* *meliloti* and *Mesorhizobium* *loti* bacteria residing in the root nodules of *M. truncatula* and *L. japonicus*, respectively, adjust their gene expression in response to water shortage. Although our results demonstrated that both *M. truncatula* and *L.* *japonicus* root nodules were susceptible to water deprivation, they indicated significant differences in plant and bacterial response to drought between the tested species, which might be related to the various types of root nodules formed by these species.

## 1. Introduction

Plants belonging to the Fabaceae family are unique due to their ability to form symbiotic interactions with nitrogen-fixing bacteria, called rhizobia. As a result of this interaction, plants receive from bacteria atmospheric dinitrogen reduced into ammonia, which, in contrast to free nitrogen, is suitable for utilisation. Such symbiosis enables fabaceans to reach a high protein content in their tissues, irrespectively of nitrogen soil resource [1]. During the establishment of symbiosis, rhizobia inhabit roots and trigger the development of specific plant organs—root nodules [2]. The aim of root nodule formation is to ensure the microaerobic environment, which is essential for the proper activity of nitrogenase—the key enzyme enabling dinitrogen fixation.

Taking into consideration the root nodule meristematic activity, there are two types of root nodules—determinate and indeterminate. The first is characterised by the loss of meristematic activity shortly after its initiation and spherical shape. In the second, an active meristem can be detected during the whole root nodule’s life. *Medicago truncatula* and *Lotus japonicus* are model plants for studying different types of root nodules [1]. The symbiosis of *M. truncatula* with its compatible strain *Sinorhizobium meliloti* results in the formation of indeterminate nodules [3], whereas *L. japonicus* develops determinate type root nodules, resulting from symbiosis with *Mesorhizobium loti* [4].

Due to climate change, water deficit is one of the major concerns of modern agriculture. Since protein-rich fabaceans are one of the most important groups of food and feed crops, there is a crucial need to discover new methods for their drought tolerance improvement [5]. Moreover, there is strong evidence that water deprivation affects nitrogen fixation in the root nodules of Fabaceae plants [6,7] and significantly limits their yield [5,8]. Drought stress drastically changes water relations and osmotic potential, and thus triggers plant stress response at many levels—molecular, cellular and physiological [9,10]. It has been shown that severe drought stress in *M. truncatula* results in oxidative tissue damage [11] and a decrease in stomatal conductance, which is one of the mechanisms to avoid water loss, but at the same time affects the efficiency of CO_2_ fixation [12]. However, osmotic adjustment [13] and increased cell membrane integrity observed under water deficit conditions allows *M. truncatula* to maintain a sufficient level of CO_2_ fixation [12]. Interestingly, it has been reported that *M. truncatula* plants exposed to drought initiate nodule senescence very quickly, even before their growth is affected, which can be a strategy to utilise the remaining nitrogen and nutrients released and recovered during senescence [14]. Another known response to drought characteristic for fabaceans is the accumulation of abscisic acid (ABA) and proline [11,15], facilitating osmoregulation [8,15,16]. It has been shown that drought stress has a negative impact on fabacean seed development and composition [17]. In *L. japonicus*, water deficit causes an increase in the soluble small molecules content [18] and global reprogramming of main metabolic pathways resulting in rearrangements within the cell wall, hormonal metabolism, synthesis of protective molecules, carbon and amino acid metabolism [19]. There are some indications that fabaceans differing in the type of nodules can have a different tolerance to drought, but, at the same time, irrespectively of the type, nodules demonstrate similar strategies for coping with stress, which has been revealed in the proteomic analysis [20]. In contrast, there is evidence for divergent strategies of salt tolerance between *M. truncatula* and *L. japonicus* [21]. Interestingly, nodulated *M. truncatula* plants recover more effectively from drought, compared to non-nodulated controls [22].

In this research, apart from showing differences in global gene expression between root and root nodules, we showed early transcriptomic changes in *M. truncatula* and *L. japonicus* root nodules caused by water deprivation. Our approach is unique because the analysis includes both plant and bacterial symbiotic partners. Only by analysing and comparing drought response of both plant and bacterial species, it is possible to explain whether the differences in drought tolerance depend on the plant or bacterial response, or both.

## 2. Results and Discussion

### 2.1. Two- and Four-Day Long Water Deprivation Results in Decreased Nitrogenase Activity and Increased Expression of Drought Stress Markers in M. truncatula and L. japonicus Root Nodules

**Figure 1:**
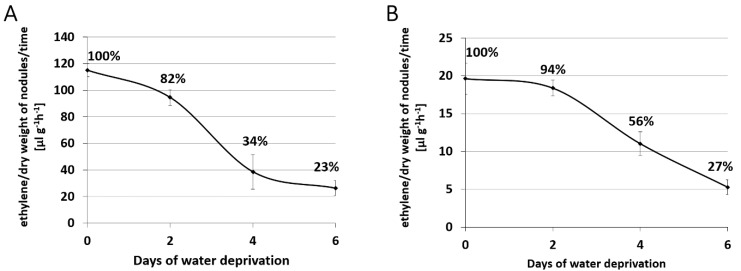
Nitrogenase activity in root nodules harvested from drought-treated *M. truncatula* (**A**) and *L. japonicus* (**B**), estimated using an acetylene reduction assay.

In order to select a time point of water deprivation, which causes initial and more severe changes within root nodules, an acetylene reduction assay [23] was performed on detached root nodules. This method is commonly used to estimate the activity of nitrogenase [24,25]. Generally, root nodules of *M. truncatula* seem to have higher nitrogenase activity in comparison to the *L. japonicus* (Figure 1). In *M. truncatula* root nodules, an 18% decrease in nitrogenase activity was noted after two days of watering withdrawal, and 66% after four days (Figure 1A). At day six without watering, the nitrogenase reached only 23% of activity observed in well-watered control *M. truncatula* root nodules (Figure 1A). Similar results were previously described for the indeterminate-type root nodules of *Pisum sativum* [6]. It has also been shown that a drought-related decline in nitrogenase activity is local, rather than systemic in both *P. sativum* and *M. truncatula*, since it occurs only in the part of root system exposed to water deprivation [6,26].

A similar tendency was noticed for *L. japonicus*; however, in this case, the decline in nitrogenase activity was slower. Two days after watering withdrawal, only a 6% decrease in nitrogen activity was observed compared to the control, while after four and six days, the value reached 56% and 27% of initial, control activity, respectively (Figure 1B). A similar drought-related decline in nitrogenase activity has repeatedly been reported for the determinate type root nodules in *Glycine max* [25,27,28].

**Figure 2:**
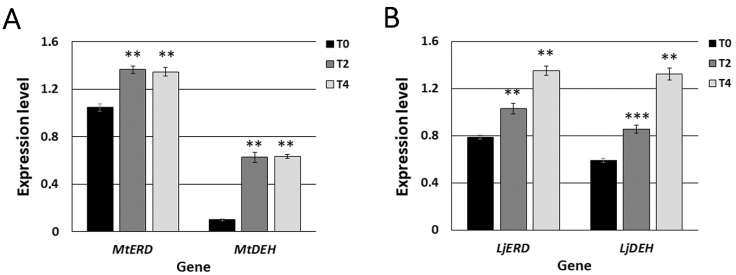
Normalised expression levels of drought stress marker genes in *M. truncatula* (**A**) and *L. japonicus* (**B**) root nodules harvested from plants grown in control conditions (T0) and after two (T2) and four (T4) days without watering. Mean values (±SE) are derived from two biological replicates, for which three individual qPCR reactions were performed (*n* = 6). Asterisks above the bars represent statistically significant differences at the level *p* < 0.01 (**) or *p* < 0.001 (***).

In order to confirm that root nodules of both *M. truncatula* and *L. japonicus* exhibited signs of drought stress, we also analysed the expression level of selected plant marker genes for early response to water deprivation: *early-responsive to dehydration stress* (*ERD*) and *dehydrin* (*DEH*). These genes had previously been shown to be highly expressed in the roots of *M. truncatula* exposed to drought [10]. In our study, the expression levels of *MtERD* and *MtDEH* were significantly higher in *M. truncatula* root nodules after only two days of water deprivation (T2), compared to the root nodules harvested from well-watered control plants (T0) (Figure 2A). Moreover, their high expression level remained constant after four days of water deprivation (T4) (Figure 2A). Similar to *M. truncatula*, the expression levels of both *LjERD* and *LjDEH* were elevated in *L. japonicus* root nodules exposed to two-day long drought stress (T2), compared to the control (T0) (Figure 2B). However, in *L. japonicus*, both marker genes demonstrated higher expression levels four days after cessation of watering (T4) than after two days (Figure 2B), which could indicate its better adaptation and greater tolerance towards water shortage stress.

### 2.2. Symbiosis with Rhizobia Drastically Changes Plant Transcriptome in both M. truncatula and L. japonicus

In order to better understand the molecular mechanisms underlying differences in the plant transcriptome caused by symbiosis, we performed RNA-Seq analysis for both root nodules and non-inoculated roots of *M. truncatula* and *L. japonicus*.

**Figure 3:**
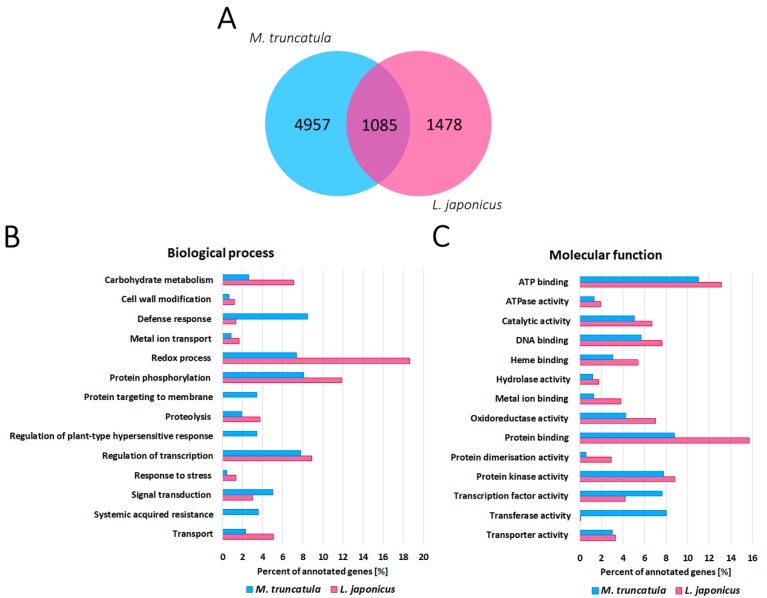
The number of genes differentially expressed in *M. truncatula* (blue) and *L. japonicus* (pink) root nodules, in comparison to the non-inoculated roots (**A**). The categorisation of differentially expressed genes in root nodules, in comparison to non-inoculated roots of *M. truncatula* (6042 genes) and *L. japonicus* (4830 genes), based on biological processes (**B**) and molecular functions (**C**).

In *M. truncatula*, we revealed 6042 genes with at least a two-fold statistically different expression level in root nodules, in comparison to the non-inoculated roots (Supplementary Data Set 1). The most elevated expression level in root nodules, compared to roots, was demonstrated by genes encoding nodulation-specific secretion proteins (Table S1), mostly nodule-specific cysteine-rich peptides (NCR), which are known to be involved in final bacteroid differentiation [29]. It is also known that cells containing bacteroids progressively increase their volume along with endocytosis-like uptake of rhizobia from infection threads and successive host cell differentiation [3,30], which could be the reason why genes participating in cell wall rearrangement were differentially expressed in the root nodules of *M. truncatula* when compared to the non-inoculated roots (Figure 3B). Furthermore, genes encoding enzymes involved in the synthesis, metabolism and degradation of secondary metabolites, especially flavonoids, exhibited differential expression. Flavonoids are a component of molecular dialogue between host plants and rhizobia and are secreted during initial stages of symbiosis, allowing activation of *nod* genes [31]. Moreover, flavonoids influence auxin transport and thus regulate local inhibition of auxin accumulation during nodulation [32]. In our study, we identified a high number of differentially expressed genes that were involved in auxin metabolism, transport and signal transduction, which might be connected with constant meristematic activity ongoing in the indeterminate root nodules [33].

In the *L. japonicus* root nodule transcriptome, we identified 4830 plant genes with at least a two-fold statistically different expression level in root nodules, in comparison to the non-inoculated roots (Supplementary Data Set 1). One of the groups of highly expressed genes in the root nodules was one encoding metal transporters (Figure 3B, Table S2), which could be associated with iron and molybdenum transport into the nodules for the composition of nitrogenase active centres [34].

Furthermore, genes encoding homocitrate synthase, involved in the synthesis of the nitrogenase FeMo cofactor, were highly expressed in the root nodules of *L. japonicus* [34,35]. Functional analysis demonstrated that genes encoding enzymes, involved in reorganising the cell wall (Figure 3B) and secondary metabolism (especially flavonoids), were differentially expressed in *L. japonicus* root nodules, similar to *M. truncatula*.

In order to identify common features for both types of root nodules, and distinctions in their metabolism, we compared transcriptomic changes in root nodules, compared to the non-inoculated roots for both species (Figure 3). For only 53% of differentially expressed *L. japonicus* genes, we were able to find orthologs within the *M. truncatula* genome (Figure 3A). Gene ontology analysis of differentially expressed genes performed for both species showed some common biological processes essential for both species, such as regulation of transcription, protein phosphorylation and redox processes (Figure 3B). However, there were also processes, which were specific only for *M. truncatula* root nodules, such as protein targeting to membrane, regulation of plant-type hypersensitive response and systemic acquired resistance (Figure 3B). Molecular functions of differentially expressed genes were quite similar for both species (Figure 3C), yet still, some minor differences could be found. For instance, we noted a much higher percentage of genes participating in protein and metal ion binding in *L. japonicus* root nodules than in *M. truncatula*, while there was a much lower percentage of genes encoding proteins with transferase activity (Figure 3C).

Of the identified orthologous genes, 1085 were commonly deregulated in the root nodules of both species, in comparison to the roots (Figure 3A). The relatively small number of commonly deregulated genes and observed differences in some gene ontologies indicate the dissimilar metabolism of *M. truncatula* and *L. japonicus* root nodules, which is probably related to their differing types.

### 2.3. RNA-Seq Analysis Revealed a High Number of Differentially Expressed Plant Genes during Water Deprivation Stress in the M. truncatula and L. japonicus Root Nodules

**Figure 4:**
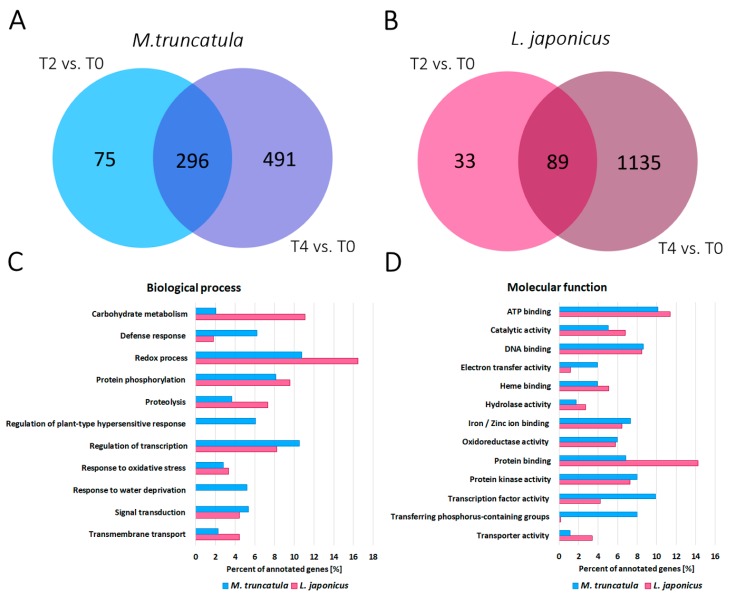
The number of *M. truncatula* (**A**) and *L. japonicus* (**B**) genes differentially expressed in root nodules during two- (T2) and four-day long (T4) drought stress, in comparison to control plants not exposed to water deprivation (T0). The categorisation of differentially expressed genes in drought-stressed (both T2 and T4) root nodules, in comparison to well-watered controls for *M. truncatula* (862 genes) and *L. japonicus* (1257 genes), based on biological processes (**C**) and molecular functions (**D**).

In the next step, we compared the transcriptome of *M. truncatula* root nodules exposed to two (T2)- and four (T4)-day long drought stress with the transcriptome of root nodules derived from plants grown in control conditions (T0) (Supplementary Data Set 1). This analysis revealed 371 and 787 genes with at least a two-fold statistically different expression level after two or four days without watering, respectively (Figure 4A). Genes with the highest and the lowest expression level in T4 are presented in Table S3. Among all the genes differentially expressed in T2 and T4, 296 were commonly deregulated. Two and four days after watering, we observed the down-regulation of numerous genes encoding transporters of sugars, amino acids or oligopeptides, which might indicate that *M. truncatula* plants provided fewer compounds for bacteroids during drought stress [35]. Four days after water cessation, genes encoding Krebs cycle’s enzymes were also down-regulated, which suggested a deficiency of substrates for cell respiration. Since the supply of carbon compounds, such as Krebs cycle intermediates, is also necessary for fixed nitrogen assimilation [36], this could be one of the reasons for less-effective nitrogen fixation during drought stress. The elevated expression level of genes encoding DNase I and some other endonucleases in both T2 and T4 is another indication of intensified nodule senescence during drought stress [37]. The same applies to the decreased expression level of genes encoding mitogen-activated protein kinases (MAPKs), regulating cell divisions [38], in both T2 and T4, as well as increased expression of genes encoding cyclin A2 in T4, which negatively regulates endoreduplication cycles [39]. Moreover, in T2, we observed a decrease in the expression level of genes involved in cellulose synthesis and cell wall rearrangement, which progressed along with the duration of water deprivation. This may indicate the slow-down in the biosynthesis of new infection threads [40]. Furthermore, in both T2 and T4, there was also a down-regulation of genes encoding enzymes responsible for biosynthesis of some secondary metabolites, such as terpenes, lignins, flavonoids and glucosinolates, many of which are necessary for correct plant-microsymbiont communication [41]. Moreover, many genes involved in hormone metabolism were deregulated. Higher expression of drought stress concerned genes encoding enzymes engaged in abscisic acid (ABA) biosynthesis and signal transduction, which might be connected with osmotic changes occurring within root nodules during water deprivation stress [42]. Meanwhile, a lower expression level characterised genes encoding enzymes responsible for hydrolysis of auxin-amino acid conjugates, jasmonic acid (JA) and ethylene (ET) biosynthesis and signal transduction (e.g., 1-aminocyclopropane-1-carboxylic acid (ACC) synthase). Both JA and ET participate in plant response towards a broad range of stresses [43,44]; therefore, such changes could positively affect plant tolerance to drought stress [44].

As in the case of *M. truncatula*, the transcriptome of root nodules, harvested from *L. japonicus* plants exposed to water deprivation for two (T2) or four (T4) days, was compared to the transcriptome of nodules derived from plants grown in control conditions (T0) (Supplementary Data Set 1). The analysis revealed 122 and 1224 differentially expressed genes after two or four days without watering, respectively (Figure 4B). In Table S4, we present the most deregulated *L. japonicus* genes after four days of water deprivation. Out of all the differentially expressed genes in T2 and T4, 89 were commonly deregulated. In both T2 and T4, we observed decreased expression levels of genes encoding proteins from the KEULE family, which are involved in vesicle transport during cytokinesis [45]. The most highly expressed gene in *L. japonicus* root nodules in T4 encoded the thaumatin-like protein (Table S4), which is drought-inducible and participates in plant adaptation to osmotic stress [46,47]. Similar to *M. truncatula*, in *L. japonicus* root nodules, we observed the down-regulation of genes encoding Krebs cycle enzymes and genes regulating the cell cycle [36]. However, *L. japonicus* genes encoding DNase I were down-regulated during drought stress; thus, together with up-regulation of genes encoding chitinases and cellulose synthase, this indicates their opposite reaction towards drought stress, in comparison to *M. truncatula*. A number of genes involved in hormone metabolism and signal transduction, dependent on plant hormones, were differentially expressed in T2 and T4, in comparison to T0. Similar to *M. truncatula* root nodules, genes encoding the key enzyme in ABA biosynthesis showed elevated expression levels in both T2 and T4 [42,48]. Moreover, genes encoding proteins induced by a high concentration of ABA and drought stress, such as phosphatase 2C, which acts as a negative regulator of ABA response [49], were also up-regulated. Meanwhile, genes contributing to brassinosteroids and ET biosynthesis, including ACC synthases, were up-regulated in T4 [43,44], which was different from the results obtained for *M. truncatula* root nodules. Elevated expression level during water deprivation stress was also a feature of genes encoding late embryogenesis abundant (LEA) glycoproteins (Table S4), which are known to be drought stress-inducible [50] and serine-rich proteins, which are induced by a variety of abiotic stresses [51]. Differential expression during drought stress was also shown by genes encoding transcription factors (Figure 4C,D) belonging to MYB [52], basic Helix-Loop-Helix, Constance-like, basic region/leucine zipper motif (bZIP) [53], WRKY, C2H2-type zinc finger and NAC families [54]. Furthermore, after four days of water deprivation, we noted up-regulation of a large number of genes encoding transcription factors from the ethylene response factor (ERF) subfamily of AP2 (AP2/ERF), which are known to be involved in plant response to drought stress [55]. Similar to *M. truncatula*, four days after watering cessation, there was deregulation of genes encoding proteins involved in cell wall remodelling, microfibril organisation as well as cellulases, glucuronidases, lyases, esterases, expansins and pectins depolymerases. In T2 and T4, we observed deregulation of genes encoding enzymes involved in the synthesis of terpenoids, lignins and flavonoids, some of which are necessary for correct plant-rhizobia communication [41]. After four days of water deprivation, we observed increased expression of genes encoding precursors of Kunitz-type protease inhibitor and cysteine proteinases (Table S4), which are involved in programmed cell death (PCD) [56,57,58]. In T4, there were also some molecular symptoms of nodule senescence, such as increased expression level of six orthologs of *Arabidopsis thaliana* gene encoding serine protease SAG12 (senescence-associated gene 12) [59] and three orthologs of SRG1 (senescence-related gene 1).

Gene ontology analysis performed for differentially expressed genes during drought for both species (Figure 4C,D) revealed changes in certain common biological processes in *M. truncatula* and *L. japonicus* root nodules, such as redox processes, protein phosphorylation and regulation of transcription (Figure 4C). However, there were also some specific features of drought-stressed *M. truncatula* nodules, such as regulation of plant-type hypersensitive response (Figure 4C). Interestingly, for both species, we detected differential expression of genes involved in response to oxidative stress, but only *M. truncatula* nodules exhibited differential expression of genes participating in response to water deprivation (Figure 4C). This observation, along with a more severe decrease in nitrogenase activity (Figure 1A), may indicate that *M. truncatula* plants are more susceptible to drought stress than *L. japonicus*.

Comparing the reactions of *M. truncatula* and *L. japonicus* towards drought stress, we found only 38 direct orthologs within genes expressed differentially in both T2 and T4. Moreover, more than half of these were regulated in the opposite manner. Although these two species seem to share some common drought-induced pathways, they seem to enact different strategies during the drought stress response, which could have its basis in their different types of root nodules (determinate vs. indeterminate). Our study was focused on the early response to drought, and at the tested time points (T2 and T4), there were no visible changes in the morphology of drought-treated plants, including both shoot and root. Therefore, we extended water deprivation until the tenth day after water cessation (T10) to check for phenotypic and microscopic changes in the nodule structure. Even after ten days of watering withdrawal, there were no morphological changes in either *M. truncatula* or *L. japonicus*. Root nodules of both species demonstrated typical anatomy (Figures S1–S4) [3,4]. Changes in the histological structure of *M. truncatula* root nodules in T10, examined by means of the light microscopy, included loss of turgor of the cortical cells, vascular endodermis cells, bundle pericycle cells, vascular parenchyma cells positioned between the tracheary elements and bacteroid tissue (Figure S2). Mitoses were visible even 10 days after watering cessation in the meristem of *M. truncatula* root nodules, and the saprotrophic zone was not yet formed in T10 nodules. Within *L. japonicus* T10 nodules, there was a shrinkage of the inner layer of the cells of the nodule cortex (Figure S4). Moreover, cortical endodermis cells within the lenticel were partially collapsed. Furthermore, all cells of the nodule cortex, as well as the vascular endodermis cells, were non-turgid, and only parenchymatic cells within the vascular bundle did not conform to this change. Bacteroid tissue was similar as in the T0 nodules (Figure S3), but the loss of turgor was noticeable in both the infected and non-infected cells. These results suggest that although both fabaceans have evolved divergent strategies of molecular adaptation to water deficiency, both of them are highly effective since no severe changes within nodules of both tested species were noticed even after ten days of water deprivation.

### 2.4. RNA-Seq Analysis Revealed Differentially Expressed Bacterial Genes during Water Deprivation Stress in the M. truncatula and L. japonicus Root Nodules

**Figure 5:**
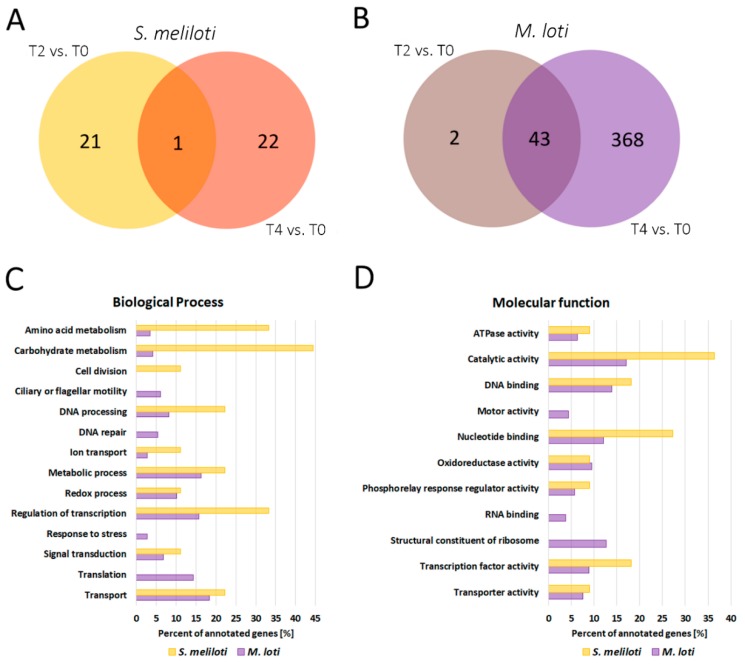
The number of *S. meliloti* (**A**) and *M. loti* (**B**) genes differentially expressed in root nodules during two- (T2) and four-day long (T4) drought stress in comparison to the control plants not exposed to water deprivation (T0). The categorisation of differentially expressed genes in drought-stressed (both T2 and T4) bacteroids, in comparison to well-watered controls for *S. meliloti* (44 genes) and *M. loti* (413 genes), based on biological processes (**C**) and molecular functions (**D**).

RNA-Seq analysis performed for nodules subjected to drought stress also revealed changes in transcriptomes of symbiotic bacteria inhabiting root nodules (Supplementary Data Set 1). In the case of *S. meliloti*, these changes were rather subtle; thus, we applied less restrictive parameters of logarithmic FC (fold change), that is, 1 < FC < −1. Genes with the highest and the lowest expression levels in T4 are presented in Table S5. We found 22 and 23 genes with different expression levels in T2 and T4, respectively, in comparison to T0. Only one of these was common to both time points of drought stress. Most of the differentially expressed genes in T2 had elevated expression levels in T2 when compared to the control T0. Among these, we distinguished genes encoding enzymes involved in bacterial DNA replication—DNA polymerase III subunit alpha and DNA topoisomerase, which is engaged in the relaxation of positively supercoiled DNA if a short single-stranded loop is formed [60,61]. A higher expression level in T2 was also a feature of glycogen debranching enzymes, which mobilise glucose reserves from glycogen [62]. This may indicate that drought-stressed bacteroids receive less carbon compound from photosynthesis. Another result suggesting bacteroid starvation is the up-regulation of genes encoding RelE toxin activated in response to nutritional stress [63] and cleaving mRNA codons positioned at the ribosomal A-site [64]. Toxin-antitoxin loci function as elements of bacterial stress response that help them to cope with starvation, possibly by preventing the production of defective proteins during stress conditions [65]. Moreover, after four days of water deprivation, the expression level of gene encoding putative sugar efflux transporter was decreased, in comparison to the control conditions. Such transporters have a broad spectrum of substrates, but generally, facilitate the efflux of sugar compounds from bacteria [66]. Therefore, it is reasonable that in conditions of carbon compounds limitation, the expression of this transporter was inhibited. Also, in T2, we observed a higher expression level of two genes encoding histidine kinases. Since histidine kinases are the part of stress signal transduction pathway, this may suggest that bacteroids suffer from water deprivation stress as a result of changes in osmolarity and turgor [67]. Another indication of a disturbance in ionic homeostasis was the lower expression level of the *S. meliloti* gene encoding Na^+^/H^+^ antiporter [68] in T2 vs. T0. We also observed three up-regulated genes encoding transcription regulators. One of these belongs to the MarR (multiple antibiotic resistance regulator) family, which is deregulated in response to specific environmental stimuli, especially those in the form of phenolic compounds, which often have plant origin [69]. Although some of the members of MarR family have been shown to be connected with a response to environmental stresses, especially those generated by antibiotic treatment [70], our data may indicate that members of the MarR family can be universal stress-responsive proteins. Moreover, two other genes encoding members of the MarR family were differentially expressed after four days of water deprivation. Another transcription regulator which was up-regulated in T2 belongs to the LysR family. Members of this family regulate oxidative stress response [71], nitrogen fixation [72] and response to nitrogen limitation [71]. Furthermore, after four days of water deprivation, we observed an increase in expression levels of two genes belonging to the phenylalanine/tyrosine degradation pathway. Phenylalanine serves as a nitrogen source under nitrogen starvation conditions [73]. It is a part of a well-known strategy to survive starvation since the removal of abnormal proteins and retrieval of amino acids from degraded ones provides compounds for the synthesis of new, critically needed proteins [73,74]. Nitrogen starvation of *S. meliloti* bacteroids could be the result of reduced nitrogenase activity, observed in *M. truncatula* root nodules in T2 and T4 (Figure 1A). In T4, we also detected a decrease in the expression level of genes encoding some proteins participating in the transport of amino acids, which is essential in the compound exchange between bacteria and plant during symbiosis [75] (Figure 5C). Down-regulation of two genes, which encode bacterial cell division proteins, was also noted (Figure 5C). This could indicate that, along with drought stress, rhizobia located in infection threads cease divisions [76], which also corresponds well with the down-regulation of *M. truncatula* genes involved in infection thread biosynthesis during drought stress. In T4, there was also down-regulation of gene encoding pilus assembly protein, which can directly affect the effectivity of symbiosis establishment [77,78], and a gene encoding nitrite reductase, which has previously been proven to inhibit activity during water deficit in bacteroids of *Glycine max* [79]. Our data indicate that water stress not only affects enzyme activity but also influences its gene expression.

RNA-Seq analysis performed for nodules subjected to drought stress also revealed changes in transcriptomes of symbiotic bacteria inhabiting root nodules (Supplementary Data Set 1). In the case of *S. meliloti*, these changes were rather subtle; thus, we applied less restrictive parameters of logarithmic FC (fold change), that is, 1 < FC < −1. Genes with the highest and the lowest expression levels in T4 are presented in Table S5. We found 22 and 23 genes with different expression levels in T2 and T4, respectively, in comparison to T0. Only one of these was common to both time points of drought stress. Most of the differentially expressed genes in T2 had elevated expression levels in T2 when compared to the control T0. Among these, we distinguished genes encoding enzymes involved in bacterial DNA replication—DNA polymerase III subunit alpha and DNA topoisomerase, which is engaged in the relaxation of positively supercoiled DNA if a short single-stranded loop is formed [60,61]. A higher expression level in T2 was also a feature of glycogen debranching enzymes, which mobilise glucose reserves from glycogen [62]. This may indicate that drought-stressed bacteroids receive less carbon compound from photosynthesis. Another result suggesting bacteroid starvation is the up-regulation of genes encoding RelE toxin activated in response to nutritional stress [63] and cleaving mRNA codons positioned at the ribosomal A-site [64]. Toxin-antitoxin loci function as elements of bacterial stress response that help them to cope with starvation, possibly by preventing the production of defective proteins during stress conditions [65]. Moreover, after four days of water deprivation, the expression level of gene encoding putative sugar efflux transporter was decreased, in comparison to the control conditions. Such transporters have a broad spectrum of substrates, but generally, facilitate the efflux of sugar compounds from bacteria [66]. Therefore, it is reasonable that in conditions of carbon compounds limitation, the expression of this transporter was inhibited. Also, in T2, we observed a higher expression level of two genes encoding histidine kinases. Since histidine kinases are the part of stress signal transduction pathway, this may suggest that bacteroids suffer from water deprivation stress as a result of changes in osmolarity and turgor [67]. Another indication of a disturbance in ionic homeostasis was the lower expression level of the *S. meliloti* gene encoding Na^+^/H^+^ antiporter [68] in T2 vs. T0. We also observed three up-regulated genes encoding transcription regulators. One of these belongs to the MarR (multiple antibiotic resistance regulator) family, which is deregulated in response to specific environmental stimuli, especially those in the form of phenolic compounds, which often have plant origin [69]. Although some of the members of MarR family have been shown to be connected with a response to environmental stresses, especially those generated by antibiotic treatment [70], our data may indicate that members of the MarR family can be universal stress-responsive proteins. Moreover, two other genes encoding members of the MarR family were differentially expressed after four days of water deprivation. Another transcription regulator which was up-regulated in T2 belongs to the LysR family. Members of this family regulate oxidative stress response [71], nitrogen fixation [72] and response to nitrogen limitation [71]. Furthermore, after four days of water deprivation, we observed an increase in expression levels of two genes belonging to the phenylalanine/tyrosine degradation pathway. Phenylalanine serves as a nitrogen source under nitrogen starvation conditions [73]. It is a part of a well-known strategy to survive starvation since the removal of abnormal proteins and retrieval of amino acids from degraded ones provides compounds for the synthesis of new, critically needed proteins [73,74]. Nitrogen starvation of *S. meliloti* bacteroids could be the result of reduced nitrogenase activity, observed in *M. truncatula* root nodules in T2 and T4 (Figure 1A). In T4, we also detected a decrease in the expression level of genes encoding some proteins participating in the transport of amino acids, which is essential in the compound exchange between bacteria and plant during symbiosis [75] (Figure 5C). Down-regulation of two genes, which encode bacterial cell division proteins, was also noted (Figure 5C). This could indicate that, along with drought stress, rhizobia located in infection threads cease divisions [76], which also corresponds well with the down-regulation of *M. truncatula* genes involved in infection thread biosynthesis during drought stress. In T4, there was also down-regulation of gene encoding pilus assembly protein, which can directly affect the effectivity of symbiosis establishment [77,78], and a gene encoding nitrite reductase, which has previously been proven to inhibit activity during water deficit in bacteroids of *Glycine max* [79]. Our data indicate that water stress not only affects enzyme activity but also influences its gene expression.

Changes in the expression of *M. loti* genes were more evident, which is why we could apply more restrictive parameters of differentially expressed genes selection (2 < FC < −2). We found 45 differentially expressed genes after two days of watering cessation and 411 after four days. Moreover, 43 genes were common for both time points. As in the case of *S. meliloti*, most of the genes (41 out of 45) differentially expressed in T2 were up-regulated. The twenty most up- and down-regulated *M. loti* genes in T4 are presented in Table S6. Similar to *S. meliloti*, in T2 and T4, we observed up-regulation of the gene encoding the glycogen debranching enzyme, which mobilises glucose reserves from glycogen [62]. Moreover, in T4, we found the up-regulation of alpha-glucosidase—an enzyme which catalyses glucose release from starch and disaccharides. This could indicate that after four days of drought, bacteroids suffer from a scarcity of carbon compounds and massively mobilise glucose reserves. The higher expression of gene encoding isocitrate lyase in T2 and T4 seems to confirm this hypothesis since this enzyme is a part of glyoxylate cycle bypassing two of the decarboxylation steps in the tricarboxylic acid cycle (TCA). Bacteria use the glyoxylate cycle to utilise simple carbon compounds (C2) to satisfy carbon requirements during limitation of more complex sources, such as glucose [80]. This phenomenon has been observed in microorganisms subjected to various stress conditions [81,82,83]. Furthermore, in T4, we noted a high expression level of formate dehydrogenase gene, which is required for utilisation of one-carbon compounds [84]. In T4, we also observed down-regulation of 20 genes encoding 30S and 50S ribosomal proteins (Table S6, Figure 5C,D), which could indicate that bacteria that are receiving less carbon and nitrogen compounds, due to the reduced nitrogenase activity in drought, retarded their basal metabolic rate. We also observed a high number of down-regulated genes encoding flagellar proteins (Table S6, Figure 5C,D). It can be assumed that under conditions of limited carbon and nitrogen sources, bacteria shift their metabolism towards the production of more crucial proteins, critical for bacteroid survival, rather than proteins needed for motility. Additionally, genes involved in the carbon-phosphorus (C-P) lyase system, machinery which enables bacteria to restore phosphorus, were down-regulated [85], possibly to prevent the existing carbon compounds from degradation. Furthermore, high expression levels of genes encoding abortive infection protein and ribonucleases in T4 may be an indication of the existence of a system similar to the *S. meliloti* toxin-antitoxin system [86]. There were also clear symptoms that *M. loti* bacteroids suffered from osmotic stress resulting from water deprivation. In T4, we observed elevated expression level of gene encoding the phage shock protein, which is known to be induced during various stresses, such as osmotic shock [87], and down-regulation of gene encoding porin (Table S6), which can be influenced by both, osmotic changes and carbon source shift [88]. We also noted the increased expression of the gene encoding mechanosensitive ion channel protein over drought duration. Mechanosensitive channels respond to mechanical forces, such as osmotic and turgor pressure, by opening, which relieves pressure and prevents cells from lysis [89]. In T4, we also noted a higher expression level of genes encoding cardiolipin synthase, known to be regulated by osmotic stress in bacteria [90]. Moreover, we observed high expression levels of genes encoding trehalose-6-phosphate phosphatase, which is common in stress conditions since trehalose functions as an osmoprotectant [91,92,93]. In both T2 and T4, we observed elevated expression level of gene encoding catalase HPII, which in *S. meliloti* has been shown to be protective against H_2_O_2_, one of the reactive oxygen species (ROS) formed under stress conditions [94], while in T4, we identified the up-regulation of NADH dehydrogenase, which is also a part of bacterial protection against oxidative stress [95] and an activator of HSP90 ATPase, which is related to cell stress response [96]. Furthermore, in both T2 and T4, we also observed up-regulation of genes encoding sigma factors of RNA polymerase, which can redirect the polymerase holoenzyme to foster the expression of stress-related genes [97,98]. Similar to *S. meliloti*, in T4, we found up-regulation of several *M. loti* genes encoding histidine kinases responsible for stress signal transduction [67]. After four days of water deprivation, there was a differential expression in many genes encoding transcription regulators. As in the case of *S. meliloti*, we noted up-regulation of transcription factor belonging to the LysR family, which can be involved in response to both oxidative stress and nitrogen limitation [71,72].

Interestingly, it seems that one of the fastest bacteroid responses towards drought stress is to induce mechanisms of DNA reparation (Figure 5C) since in T4 we observed high expression levels of several ATP-dependent ligases and Ku proteins, which are required for DNA repair by non-homologous end joining of DNA double-strand breaks [99]. Possibly this very fast reaction is aimed at the prevention of long-term and fatal damage to genetic material. Moreover, the gene encoding uracil-DNA glycosylase, which takes part in the prevention of G:C→A:T transition mutations [100], was up-regulated. Quite mysterious seemed to be the high expression level of genes encoding circadian clock proteins after four days of drought. In cyanobacteria, these proteins regulate autophosphorylation cycles, nitrogen fixation, photosynthesis, amino acid uptake, gene expression and cell divisions [101]. However, their role in drought stress requires further elucidation.

RNA-Seq analysis revealed that both *S. meliloti* and *M. loti* bacteroids suffered from water deprivation, which manifests itself in osmotic changes and carbon compound limitation. Although *S. meliloti* and *M. loti* seemed to share some responses to drought, such as increased expression of histidine kinases or transcription regulators, their general strategy of survival seems to be different. In *S. meliloti*, only one differentially expressed gene was common in early (T2) and late (T4) stage of drought; hence, various genes were deregulated in tested stages of water deprivation. In *M. loti*, however, we observed the opposite situation. Only two differentially expressed genes in T2 were not detected in T4, while for the overwhelming majority of *M. loti* genes, the effect of up- or down-regulation intensified along with drought duration. Moreover, performed gene ontology analysis confirmed the existence of many differences among the species. *S. meliloti* bacteroids showed much stronger symptoms of nitrogen deficit (Figure 5C), which could result from the greater decrease in nitrogenase activity observed for *M. truncatula* nodules during drought (Figure 1A). Meanwhile, in circumstances of water deprivation, only *M. loti* bacteroids implemented some mechanisms of DNA protection (Figure 5C), down-regulated genes encoding flagellar proteins (Figure 5C,D) and possibly triggered retardation of basal metabolic rates by down-regulation of genes encoding ribosomal proteins (Figure 5C,D).

## 3. Materials and Methods

### 3.1. Plant Material, Inoculation with Rhizobia, Growth Conditions and Drought Stress Induction

Seeds of *Lotus japonicus* cv. Gifu B-129 and *Medicago truncatula* cv. Jemalong A17 were treated with 96% sulfuric acid (H_2_SO_4_) for 11 min and washed five times in sterile, de-ionised water. Subsequently, seeds were placed in Petri dishes with 1% water agar and kept in darkness at 4 °C for 12 h. After this time, Petri dishes containing *L. japonicus* seeds were placed upside-down in a growth chamber (16 h photoperiod, photosynthetic photon flux density (PPFD) of 80–100 µmol·m^−2^·s^−1^ and temperature 25 °C) for four days, and then inoculated with a *Mesorhizobium loti* strain MAFF303099 culture; whereas Petri dishes with *M. truncatula* seeds were placed in the same conditions but in normal, downside-down position and inoculated with *Sinorhizobium meliloti* strain Rm1021. Inoculum of both strains were prepared by growing bacteria on Tryptone-Yeast medium (0.5% (*w*/*v*) tryptone, 0.3% (*w*/*v*) yeast extract, 6 mM CaCl_2_, pH = 6.8) until the value of culture optical density at 600 nm (OD_600_) was between 0.6 and 0.8. Inoculated seedlings were subsequently placed in pots filled with perlite and watered with inoculum diluted 60-fold. Afterwards, pots were covered with transparent plastic foil for the next 7–10 days to protect seedlings from drying. After inoculation, plants were grown under constant conditions (16 h photoperiod, PPFD of 110–170 µmol·m^−2^·s^−1^ and temperature of 22 °C) for 6–8 weeks before harvest and watered three times per week with a carefully measured portion of nitrogen-free Fahraeus medium [102]. Plants were subjected to drought stress by cessation of watering for 2, 4, 6 and 10 days, whereas control plants were watered normally all the time until harvest. An additional non-inoculated control was grown in the same conditions, but watered with full Fahraeus medium, containing a nitrogen source [102].

### 3.2. Estimation of Nitrogenase Activity

Estimated nitrogenase activity within the nodules was measured with an acetylene reduction assay [23]. Nodules from control and drought-treated plants were detached and placed into sealed measuring vessels of 4.8 mL volume. Immediately after sealing the vessels, 0.5 mL of acetylene was injected inside. After 1 h of incubation at 25 °C, ethylene content was measured on a gas chromatograph with a mass spectrometer (GC-MS) [103]. The measurement was performed by sampling 100 µL of the gas phase from vessels in 3 technical repetitions. Subsequently, the samples were dried at 105 °C for 3 h and weighted afterwards. Estimated nitrogenase activity is presented in the chart as the volume of ethylene per nodule dry weight produced in 1 h and for both of the species. The activity observed in the control root nodules was taken as a 100% activity.

### 3.3. RNA Isolation and Sequencing

RNA was obtained from two biological replicates of *L. japonicus* and *M. truncatula* non-inoculated roots and root nodules either subjected to drought for two (T2) or four (T4) days or well-watered control nodules (T0). Total RNA extraction was performed as described previously [104]. In the case of samples derived from root nodules, an additional rRNA subunit was observed after gel electrophoresis, which proved that, along with plant RNA, bacterial RNA had been extracted (Figure S5). Isolated RNA samples were further analysed by VIB Nucleomics Core (Leuven, Belgium) using an RNA 6000 Nano Assay Kit and 2100 Bioanalyzer (Agilent Technologies, Santa Clara, CA, USA) and purified from rRNA with Ribo-Zero rRNA Removal Kits (Epicentre, Madison, WI, USA). Analysed RNA samples were used for library preparation with a TruSeq Stranded mRNA Sample Prep Kit (Illumina, San Diego, CA, USA) in two biological replicates and sequenced using NextSeq500 (Illumina, San Diego, CA, USA) with 75 base pairs length of reads and 35 million reads per run. Data obtained from RNA-Seq were normalised and annotated, then comparative analysis, based on statistically significant differences, was performed, where results were considered as significant if FDR (false discovery rate) was less than 0.05 (FDR < 0.05). Full access to the RNA-Seq data is available at the Gene Expression Omnibus (https://www.ncbi.nlm.nih.gov/geo/query/acc.cgi?acc=GSE126986). The complete list of differentially expressed genes is available in Supplementary Data Set 1. Transcripts with significantly changed expression were functionally analysed using MapMan 3.5.1R2 [105]. Gene Ontologies of differentially expressed genes were assigned by comparing our results to selected databases: Rhizobase for bacterial genes [106], Lotus Base for *L. japonicus* genes [107] and LegumeIP for *M. truncatula’s* [108]. Most abundant gene ontology categories within specific comparisons are presented in the Figures.

### 3.4. cDNA Synthesis and Real-Time qPCR

cDNA synthesis was performed as described previously [104]. Real-time qPCR was performed using the Connect CFX Real-Time PCR Detection System (Bio-Rad, Hercules, CA, USA) with Power SYBR Green Master Mix (Thermo Fisher Scientific, Waltham, MA, USA) or SsoFast EvaGreen Supermix (Bio-Rad, Hercules, CA, USA) for plant or bacterial genes, respectively. Each reaction was performed in two biological replicates and three technical repetitions. Reaction conditions are presented in Tables S7 and S8. Primers were designed with Primer3Plus [109], and their sequences are presented in Table S9. The specificity of each primer pair was verified using melting curve analysis. Reaction efficiency was calculated with the help of the LinRegPCR tool [110]. Statistical analysis of the results, which included the calculation of the relative gene expression level and the significance of the difference between tested samples, was performed using REST2009 [111]. Results were normalised using reference genes *MtFAR* (MTR_4g134960—F-box/ankyrin repeat SKIP35-like protein) for *M. truncatula* genes, *LjPP2AA2* (Lj2g3v0742070—SERINE/THREONINE-PROTEIN PHOSPHATASE PP2A) for *L. japonicus* genes and *Sm16SrRNA* (SMc03222) and *Ml16SrRNA* (MAFF_RS14480) coding 16S rRNA subunit for *S. meliloti* and *M. loti* genes, respectively. The expression level of selected genes from RNA-Seq was verified by real-time qPCR for another two biological repetitions (Table S10).

Sequences of *L. japonicus* drought stress marker genes were retrieved from the Kazusa database v. 3.0 [112,113] by comparing protein sequences of known *M. truncatula ERD* and *DEH* to *L. japonicus* proteome using the BLAST search tool [114].

### 3.5. Microscopic Analysis

Nodules were sampled from 10 plants of *M. truncatula* or *L. japonicus* at T0 (well-watered control), as well as after 10 days of watering cessation (T10) from the oldest part of the root system. The nodule surface was shallowly cut off to allow better exchange of reagents, and subjected to a routine preparation procedure, described previously [115], to obtain epoxy semi-thin (3–4 pm) sections for light microscopy examinations. Sections were stained with filtered aqueous solution of 0.1% Toluidine Blue O (Merck KGaA, Darmstadt, Germany) in 2.5% Na_2_CO_3_, pH 11.1 at 70 °C [116], mounted in DePeX (SERVA Electrophoresis GmbH, Heidelberg, Germany), and examined by means of a Provis AX70 (Olympus Corporation, Tokyo, Japan) light microscope. Digital images of 3724 × 2743 pixel resolution were saved as tiff files using a dedicated DP50 camera (Olympus Corporation, Tokyo, Japan) operating under AnalySIS software (Soft Imaging Systems GmBH, Münste, Germany). Digital images were merged—if needed—using Photoshop CS6 (Adobe Systems Inc., San Jose, CA, USA) software (panorama tool), and adjusted using the same software by means of non-destructive tools (contrast, levels, and/or curves). All adjustments were performed on the whole area of an image. Figures presenting representative nodules sampled at T0 and T10 were prepared using CorelDRAW^®^X5 software (Corel Corporation, Ottawa, ON, Canada).

## 4. Conclusions

The presented research provides a novel insight into the regulation of both bacteroid and plant transcriptomes within nodules in response to early symptoms of drought stress. Our results prove that not only plants but also rhizobia inhabiting root nodules suffer from drought stress. However, *S. meliloti* and *M. loti* demonstrate divergent responses to drought stress. Also, *M. truncatula* and *L. japonicus* apply different strategies to cope with water deficit. This phenomenon could be related to the various types of nodules they develop. However, the differences in host plant adaptive strategies could, at least partially, result from their rhizobial symbionts, exhibiting a different response to stress.

## Supplementary Materials

Supplementary materials can be found at https://www.mdpi.com/1422-0067/20/5/1204/s1.
