# Supplementary material for: Transcriptomic Changes in Medicago truncatula and Lotus japonicus Root Nodules during Drought Stress
Source: Int J Mol Sci. 2019 Mar 9;20(5):1204. doi: 10.3390/ijms20051204 (PMC6429210; doi:10.3390/ijms20051204)
Supplement: Supplementary file 1 [file ijms-20-01204-s001.zip › Supplementary Material.pdf]

Article

Transcriptomic changes in *Medicago truncatula* and *Lotus japonicus* root nodules during drought stress

Izabela Sańko-Sawczenko[1], Barbara Łotocka[1], Jakub Mielecki[2], Hanna Rekosz-Burlaga[3] and Weronika Czarnocka[1,*]

[1] Department of Botany, Faculty of Agriculture and Biology, Warsaw University of Life Sciences, Nowoursynowska 159, 02-776 Warsaw, Poland; izabela_sanko_sawczenko@sggw.pl (I.S.-S.); barbara_lotocka@sggw.pl (B.Ł.);
[2] Department of Plant Genetics, Breeding and Biotechnology, Faculty of Horticulture, Biotechnology and Landscape Architecture, Warsaw University of Life Sciences, Nowoursynowska 159, 02-776 Warsaw, Poland; jakub_mielecki@sggw.pl (J.M.);
[3] Department of Microbial Biology, Faculty of Agriculture and Biology, Warsaw University of Life Sciences, Nowoursynowska 159, 02-776 Warsaw, Poland; hanna_rekosz_burlaga@sggw.pl (H.R.-B.);
* Correspondence: weronika_czarnocka@sggw.pl; Tel.: +48-22-59-326-61

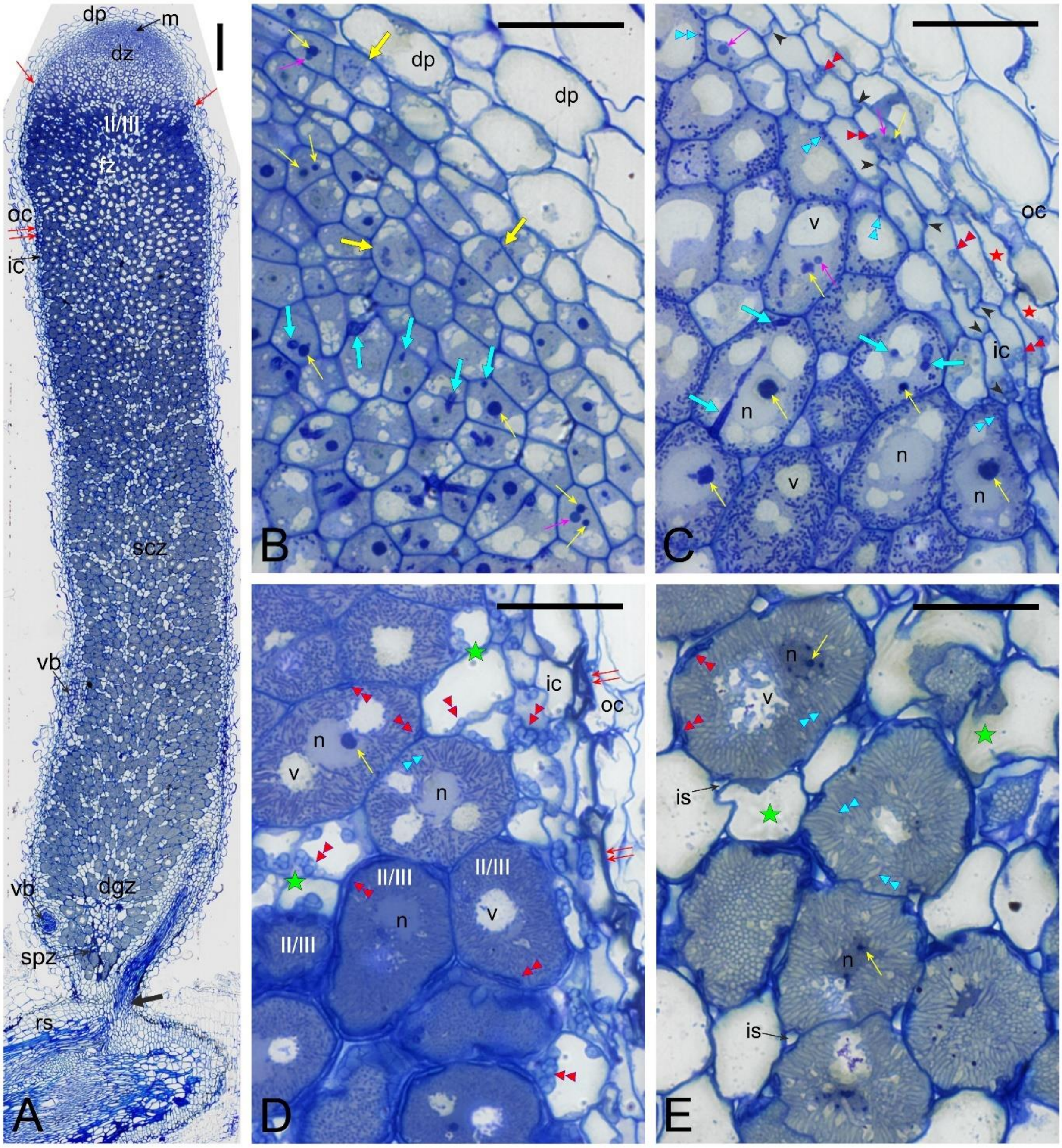

Figure S1. Structure of a representative *Medicago truncatula* root nodule sampled at T0 - from well-watered plants. Nodule central sagittal longisection showing the general anatomy (**A**). Boundary of the nodule meristem and infection zone (**B**). Nodule cortex at the mid-part of the bacteroid tissue differentiation zone (**C**). Proximal part of the differentiation zone (**D**). Bacteroid-containing tissue in the senescent zone (**E**). Labels: dp - non-specialized distal parenchyma of the nodule cortex; dgz - degraded zone; dz - differentiation zone; fz: fully-differentiated bacteroid-containing tissue; ic - inner cortical layers; m - rhizobia-free nodule meristem; n – nuclei; oc - outer cortical parenchyma; rs - root stele; scz - senescent zone; spz - saprotrophic zone; v – vacuole; vb - vascular bundles; II/III - II/III interzone; arrowheads - anticlinal walls; black arrow - vascular bundles that merged; blue arrows - infection thread apices; blue double arrowheads -cells containing symbiosomes; double red arrows - proximal part of dz; green asterisks - amyloplasts in the non-infected cells; pink asterisks - cortical endodermis; red arrows - first differentiated cells of the uniseriate cortical endodermis; red double arrowheads - collapsed cells of the cortical endodermis; red double arrowheads - large flattened amyloplasts. Bars: 200 pm (A), 50 pm (B-E).

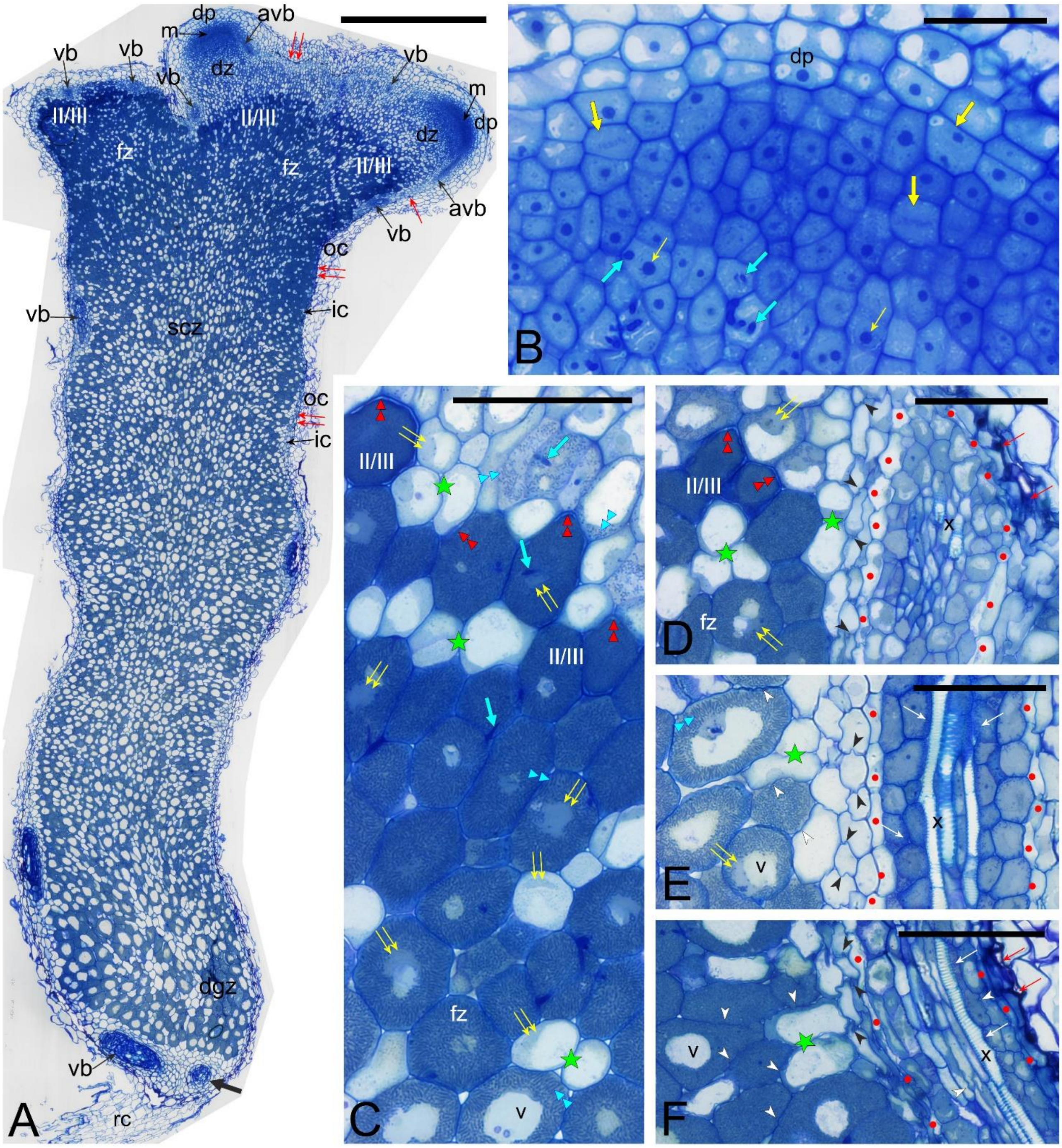

Figure S2. Structure of a representative mature *Medicago truncatula* root nodule sampled at T10. Nodule central sagittal longisection showing general anatomy (**A**). Boundary of the nodule meristem and infection zone (**B**). Central part of the bacteroid-containing tissue in the II/III interzone and dinitrogen fixation zone (**C**). Lateral part of bacteroid tissue in the II/III interzone together with the adjoining cortical tissues and vascular bundle (**D**). Lateral part of bacteroid tissue in the fixation zone together with the adjoining cortical tissues and vascular bundle (**E**). Labels: avb - apical parts of the nodule vascular bundles; dp - non-specialized distal parenchyma of the nodule cortex; dgz - degraded zone; dz - differentiation zone; fz: fully-differentiated bacteroid-containing tissue; ic - inner cortical layers; m - rhizobia-free nodule meristem; oc -outer cortical parenchyma; rc - root cortex; scz - senescent zone; v – vacuole; vb - vascular bundles; x - the tracheary elements; II/III - II/III interzone; large yellow arrows: - cytokinesis and two metaphase cells; red dots - vascular endodermis; white arrowheads - sinuous cell walls; yellow double arrows – nuclei. Bars: 200 pm (A), 25 pm (B), 50 pm (C-F).

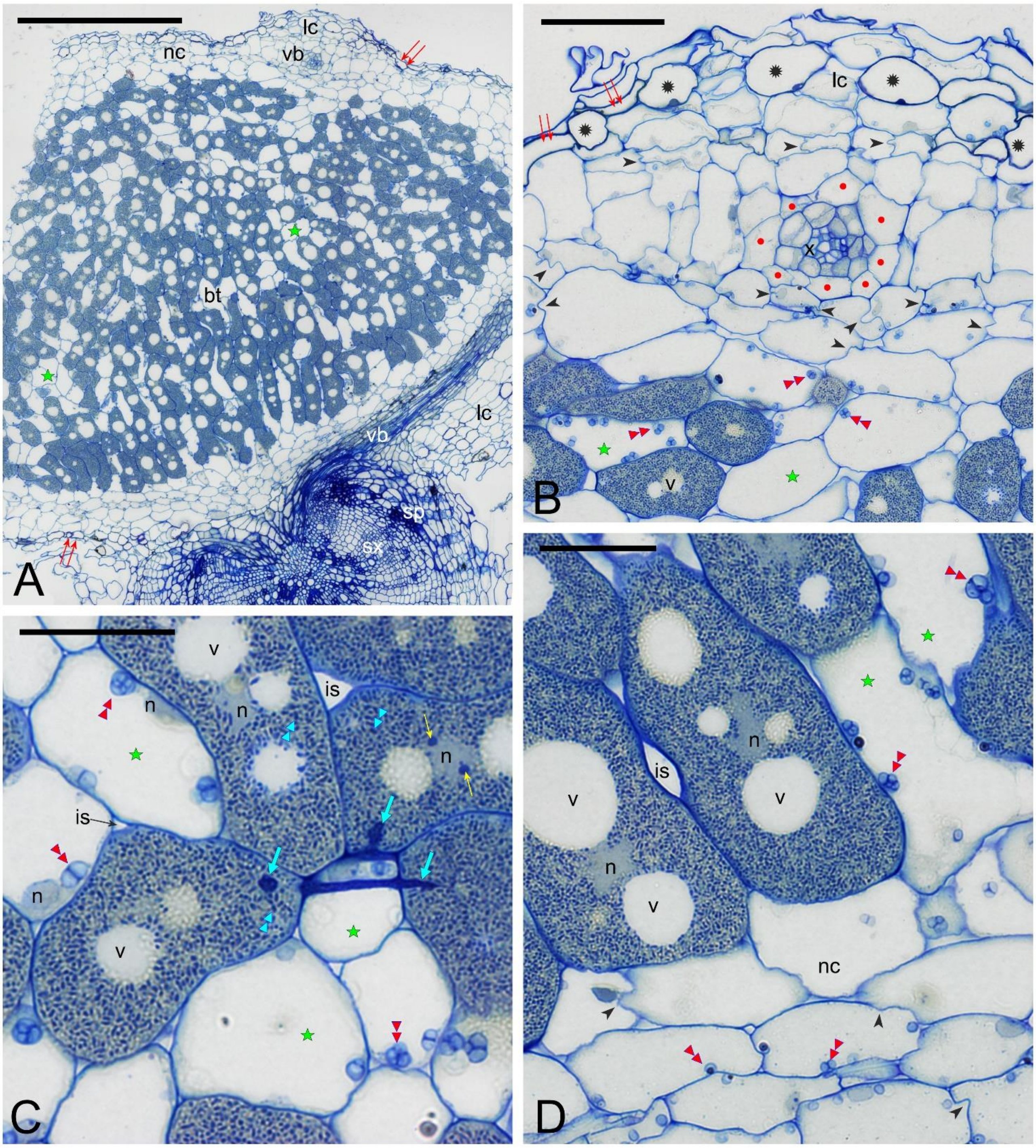

Figure S3. Structure of a representative *Lotus japonicus* root nodule sampled at T0 - from well-watered plants. Nodule central longisection (transversal vs. the root) showing general anatomy (**A**). The vascular bundle apex surrounded by nodule cortex cells (**B**). Fragments of bacteroid-containing central parenchyma at nodule apex (**C**) and base (**D**). Labels: bt - bacteroid -containing central parenchyma; is - intercellular space; lc – lenticels; n – nuclei; nc - nodule cortex; sp - secondary phloem; sx - secondary xylem; v – vacuole; vb - vascular bundles; arrowheads - sinuous cell walls; black arrowheads - sinuous cell walls; blue arrows - infection threads; blue double arrowheads - symbiosomes; double red arrows - cortical endodermis; green asterisks - non-infected cells; red double arrowheads - rounded amyloplasts; red dots - vascular endodermis; rosettes - turgid cells; thin yellow arrows – nucleoli; x - tracheary elements. Bars: 600 pm (A), l00 pm (B), 50 pm (C, D).

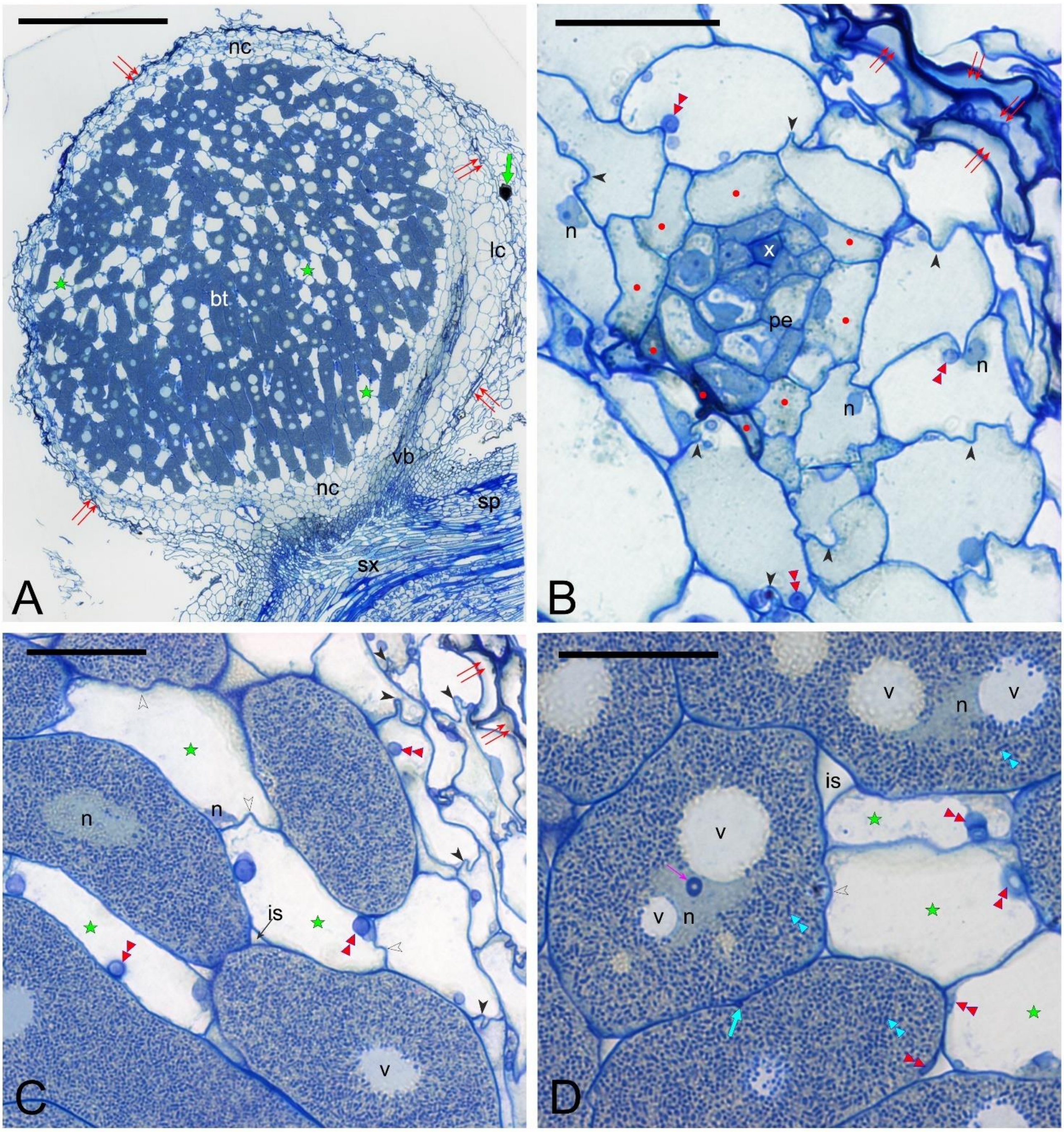

Figure S4. Structure of a representative mature *Lotus japonicus* root nodule sampled at T10. Nodule central sagittal longisection showing general anatomy (**A**). Vascular bundle apex surrounded by nodule cortex cells (**B**). Fragments of bacteroid-containing central parenchyma at nodule apex (**C**) and base (**D**). Labels: is – intercellular space; lc – lenticels; green arrow - tannin cell; n – nuclei; nc - nodule cortex; pe – pericycle; sp - secondary phloem; sx - secondary xylem; v – vacuole; vb - vascular bundles; black arrowheads – sinuous cell walls; blue double arrowheads – symbiosomes; empty arrowheads – sinuous cell walls; green asterisks - non-infected cells; red double arrows – cortical endodermis; red double arrowheads – rounded amyloplasts; thin pink arrow – Cajal body. Bars: 600 pm (A), 50 pm (B), 25 pm (C, D).

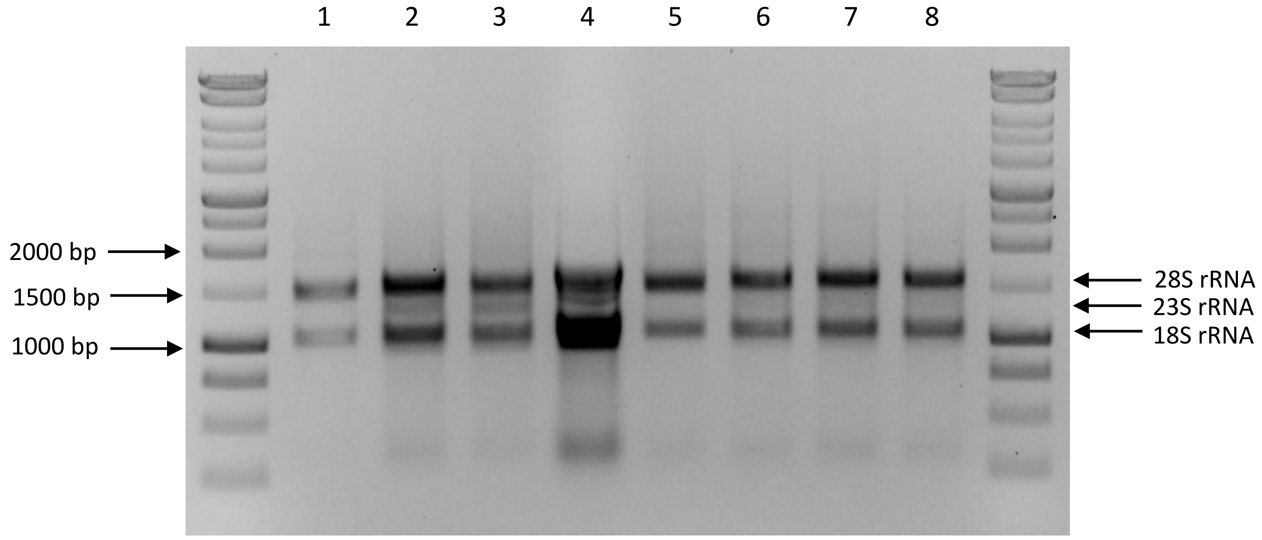

Figure S5. Electrophoretic separation of RNA samples. Wells 1–4 represent samples of RNA extracted from *M. truncatula*, while 5–8 from *L. japonicus*. Wells 1 and 5 contained RNA from roots, while 2 and 6, 3 and 7, 4 and 8 contained RNA from root nodules collected from control (T0) and drought-stressed plants (T2 and T4), respectively. bp- base pairs

Table S1. Twenty most up- and down-regulated *M. truncatula* genes in the nodules in comparison to the roots.

Gene ID	Description	log. FC
MTR_2g030470	stress up-regulated Nod 19 protein	12.50
MTR_4g065085	late nodulin	12.23
MTR_2g103313	embryo-specific protein	12.16
MTR_0416s0030	leginsulin related MtN11/16/17 family	12.06
MTR_3g031340	Nodule Cysteine-Rich (NCR) secreted peptide	11.96
MTR_4g130800	plastocyanin-like domain protein	11.79
MTR_3g052100	Nodule Cysteine-Rich (NCR) secreted peptide	11.73
MTR_4g065390	Nodule Cysteine-Rich (NCR) secreted peptide	11.71
MTR_0386s0010	Nodule Cysteine-Rich (NCR) secreted peptide	11.50
MTR_4g066160	uknown protein	11.47
MTR_1g042940	Nodule Cysteine-Rich (NCR) secreted peptide	11.26
MTR_3g020980	Nodule Cysteine-Rich (NCR) secreted peptide	11.23
MTR_3g015845	Nodule Cysteine-Rich (NCR) secreted peptide	11.18
MTR_3g464210	sulfate transporter-like protein	11.18
MTR_0337s0090	Nodule Cysteine-Rich (NCR) secreted peptide	11.18
MTR_4g017920	Nodule Cysteine-Rich (NCR) secreted peptide	11.17
MTR_4g059837	Nodule Cysteine-Rich (NCR) secreted peptide	11.13
MTR_7g029760	Nodule Cysteine-Rich (NCR) secreted peptide	11.11
MTR_7g034210	Kunitz type trypsin inhibitor / miraculin	11.10
MTR_5g041610	leghemoglobin Lb120-1	11.09
MTR_8g045640	pathogenesis-related protein bet V I family protein	-9.45
MTR_4g118610	sugar porter (SP) family MFS transporter	-9.46
MTR_1g038680	cationic peroxidase	-9.47
MTR_2g064425	monoterpene synthase	-9.68
MTR_5g017860	peroxidase family protein	-9.72
MTR_2g069300	1-aminocyclopropane-1-carboxylate oxidase-like protein	-9.84
MTR_1g092870	hippocampus abundant transcript-like protein	-9.90
MTR_2g020710	sugar porter (SP) family MFS transporter	-10.05
MTR_0018s0230	glutamate receptor 2	-10.22
MTR_4g069810	specific tissue protein	-10.27
MTR_2g044140	flavin containing monooxygenase-like protein, putative	-10.45
MTR_1g034120	gibberellin 2-beta-dioxygenase	-10.54
MTR_5g074710	peroxidase family protein	-10.79
MTR_8g028780	oxidoreductase/ferric-chelate reductase	-10.80
MTR_5g074580	orcinol O-methyltransferase	-10.93
MTR_4g114340	peptide/nitrate transporter	-11.38
MTR_5g075680	Mn-specific cation diffusion facilitator transporter MTP8.1	-11.42
MTR_3g088675	chalcone and stilbene synthase family protein	-11.82
MTR_3g009010	glycoside hydrolase family 5 protein	-12.59
MTR_8g045890	embryonic abundant-like protein	-14.09

Table S2. Twenty most up- and down-regulated *L. japonicus* genes in the nodules in comparison to the roots.

Gene ID	Description	log. FC
Lj4g3v1500410	Metal transporter	14.20
Lj1g3v0318450	PREDICTED: casein kinase I isoform delta-like	13.55
Lj3g3v0139560	Auxin-induced protein 5NG4, Nodulin MtN21 /EamA-like transporter family protein	13.30
Lj0g3v0205259	PREDICTED: bifunctional nitrilase/nitrile hydratase NIT4A-like	12.84
Lj1g3v4515810	Peptide transporter PTR3-A	12.38
Lj0g3v0205249	uknown protein	12.33
Lj0g3v0289849	cell wall protein	12.08
Lj0g3v0332609	Putative polyol transporter protein 3	12.07
Lj4g3v0336000	PREDICTED: WAT1-related protein At1g68170-like	11.74
Lj0g3v0328799	PREDICTED: ribonucleoside-diphosphate reductase small chain-like	11.68
Lj0g3v0091689	Copper transporter	11.40
Lj2g3v1729000	uknown protein	11.35
Lj1g3v3690210	uknown protein	11.34
Lj5g3v0035290	Leghemoglobin	11.30
Lj1g3v3690220	uknown protein	11.28
Lj1g3v3690250	Homocitrate synthase	11.27
Lj1g3v3690240	uknown protein	11.15
Lj1g3v3690230	Homocitrate synthase	11.14
Lj5g3v0465970	Leghemoglobin	11.12
Lj1g3v3441260	F-box/kelch-repeat protein	11.02
Lj0g3v0092569	Type IIIa membrane protein cp-wap11	-8.34
Lj3g3v0797430	Pectinesterase	-8.42
Lj0g3v0297549	Cytokinin-O-glucosyltransferase	-8.46
Lj0g3v0114299	PREDICTED: 8-hydroxyquercetin 8-O-methyltransferase-like	-8.50
Lj0g3v0200679	Pectinesterase	-8.55
Lj0g3v0085739	uknown protein	-8.59
Lj1g3v4830170	PREDICTED: chitinase 10-like	-8.61
Lj0g3v0134529	PREDICTED: MLP-like protein 34-like	-8.61
Lj4g3v2023750	Double-strand-break repair protein rad21	-8.71
Lj0g3v0285979	Sieve element occlusion by forisomes 1	-8.83
Lj0g3v0246819	Matrix metalloprotease	-8.91
Lj5g3v1604480	Pectinesterase	-8.93
Lj1g3v2067530	PREDICTED: carotenoid 9,10(9',10')-cleavage dioxygenase-like	-9.02
Lj0g3v0360999	ferric reductase	-9.04
Lj4g3v0938850	Receptor protein kinase PERK1	-9.23
Lj4g3v0189800	PREDICTED: peroxidase 7-like	-9.52
Lj0g3v0220289	PREDICTED: GDSL esterase/lipase At1g29670-like	-9.74
Lj1g3v4551230	K(+)/H(+) antiporter	-10.03
Lj3g3v0115990	uknown protein	-11.07
Lj0g3v0356449	Beta-ocimene synthase	-11.14

Table S3. Twenty most up- and down-regulated *M. truncatula* genes after four days of water deprivation.

Gene ID	Description	log. FC	
		T2 vs. T0	T4 vs. T0
MTR_1308s0010	flavin containing monooxygenase-like protein, putative	1.71	5.37
MTR_1088s0010	flavin-binding monooxygenase-like protein	1.91	5.35
MTR_4g080700	papain family cysteine protease	2.53	5.24
MTR_2g075830	papain family cysteine protease	2.68	5.22
MTR_4g028510	F-box protein interaction domain protein	4.64	5.03
MTR_6g086485	uknown protein	2.29	4.83
MTR_7g081050	triacylglycerol lipase-like protein	2.31	4.82
MTR_7g100070	type I inositol-1,4,5-trisphosphate 5-phosphatase	2.94	4.72
MTR_1g040335	Lipid transfer protein	1.76	4.57
MTR_4g109140	DUF538 family protein	2.36	4.55
MTR_4g057585	serine carboxypeptidase-like protein	2.22	4.54
MTR_4g057595	serine carboxypeptidase-like protein	2.13	4.49
MTR_4g011250	late embryogenesis abundant protein group 3 protein	3.51	4.49
MTR_2g062430	DUF946 family protein	2.32	4.47
MTR_4g107930	papain family cysteine protease	1.89	4.47
MTR_1g083180	myb-like DNA-binding domain, shaqkyf class protein	1.60	4.46
MTR_6g445520	replication factor-A carboxy-terminal domain protein	1.30	4.36
MTR_4g079770	papain family cysteine protease	1.63	4.36
MTR_4g080730	papain family cysteine protease	2.09	4.31
MTR_0001s0660	zinc-binding alcohol dehydrogenase family protein	1.06	4.24
MTR_5g094570	cytochrome P450 family 71 protein	-2.94	-5.54
MTR_3g063170	(3S)-linalool/(E)-nerolidol/(E,E)-geranyl linalool synthase	-3.13	-5.72
MTR_1g050382	zinc finger, C3HC4 type (RING finger) protein	-3.06	-5.75
MTR_0927s0010	(3S)-linalool/(E)-nerolidol/(E,E)-geranyl linalool synthase	-2.84	-5.78
MTR_2g040830	chitinase	-2.04	-5.82
MTR_5g094560	cytochrome P450 family 71 protein	-3.33	-5.86
MTR_5g094540	cytochrome P450 family 71 protein	-3.01	-5.89
MTR_6g064980	(3S)-linalool/(E)-nerolidol/(E,E)-geranyl linalool synthase	-3.68	-5.94
MTR_6g084770	cytochrome P450 family 71 protein	-3.13	-5.97
MTR_8g020990	seed linoleate 9S-lipoxygenase	-3.55	-5.99
MTR_2g007960	auxin-induced 5NG4-like protein	-4.34	-6.05
MTR_4g009590	UPF0481 plant-like protein	-4.92	-6.13
MTR_3g063120	(3S)-linalool/(E)-nerolidol/(E,E)-geranyl linalool synthase	-3.25	-6.18
MTR_3g465090	(3S)-linalool/(E)-nerolidol/(E,E)-geranyl linalool synthase	-3.26	-6.19
MTR_4g033200	isoflavone-7-O-methyltransferase	-3.25	-6.22
MTR_7g057340	(E)-beta-caryophyllene synthase	-3.76	-6.33
MTR_2g086040	cytochrome P450 family 94 protein	-3.60	-6.55
MTR_4g033015	isoflavone-7-O-methyltransferase	-3.17	-6.72
MTR_7g057390	(E)-beta-caryophyllene synthase	-3.26	-7.17
MTR_5g066690	zinc finger, C3HC4 type (RING finger) protein	-3.29	-7.21

Table S4. Twenty most up- and down-regulated *L. japonicus* genes after four days of water deprivation.

Gene ID	Description	log. FC	
		T2 vs. T0	T4 vs. T0
Lj1g3v4669300	Thaumatin-like protein	1.40	8.13
Lj3g3v2225730	PREDICTED: late embryogenesis abundant protein 1-like	1.74	7.40
Lj2g3v3292410	uknown protein	3.28	7.24
Lj2g3v0741960	uknown protein	2.22	7.08
Lj0g3v0200689	uknown protein	3.36	6.66
Lj1g3v4790770	Galactinol synthase	1.87	6.57
Lj1g3v1720110	PREDICTED: cytochrome b5-like	1.67	6.53
Lj1g3v1341630	PREDICTED: NAC domain-containing protein 19	0.92	6.51
Lj3g3v2039900	uknown protein	1.96	6.42
Lj1g3v3381270	uknown protein	1.25	6.36
Lj0g3v0346819	NAC domain protein NAC3	3.91	6.24
Lj0g3v0169979	uknown protein	0.00	6.20
Lj4g3v1534900	PREDICTED: U-box domain-containing protein 19-like	3.68	6.20
Lj1g3v3137680	Auxin-induced protein 5NG4	1.15	6.00
Lj1g3v4862910	uknown protein	2.98	5.99
Lj0g3v0000069	Cysteine proteinase	-0.71	5.97
Lj2g3v2543820	Aspartic proteinase nepenthesin-1	2.32	5.93
Lj2g3v2553830	PREDICTED: aspartic proteinase nepenthesin-1-like	2.18	5.92
Lj3g3v1641950	Cysteine proteinase	0.00	5.92
Lj4g3v0451200	CASP-like protein	0.92	5.92
Lj0g3v0037739	Type II inositol-1,4,5-trisphosphate 5-phosphatase	-2.07	-4.17
Lj0g3v0040079	PREDICTED: auxin-induced protein X10A-like	-1.64	-4.31
Lj0g3v0058079	Cytochrome P450	-0.52	-4.33
Lj0g3v0091749	uknown protein	-3.03	-4.47
Lj2g3v2902510	uknown protein	-0.96	-4.54
Lj1g3v3439010	uknown protein	-1.66	-4.60
Lj3g3v2118200	uknown protein	-1.11	-4.71
Lj1g3v4831740	PREDICTED: polygalacturonase At1g48100-like	-0.88	-4.91
Lj5g3v2220310	PREDICTED: probable pectate lyase 12-like	-1.20	-5.02
Lj2g3v2124690	PREDICTED: protein FIZZY-RELATED 2-like	-3.19	-5.09
Lj2g3v1550530	PREDICTED: long-chain-alcohol oxidase FAO4A-like	-2.14	-5.33
Lj0g3v0037719	PREDICTED: type I inositol 1,4,5-trisphosphate 5-phosphatase CVP2-like	-2.66	-5.43
Lj1g3v0052160	uknown protein	-2.25	-5.50
Lj0g3v0362339	Cytochrome P450	-1.83	-5.57
Lj0g3v0340999	PREDICTED: cyclin-U4-1-like	-2.08	-5.59
Lj0g3v0318349	uknown protein	-2.97	-5.99
Lj0g3v0081509	uknown protein	-1.26	-6.14
Lj5g3v0529330	PREDICTED: 14 kDa proline-rich protein DC2.15-like	-2.29	-6.36
Lj4g3v1037540	GAST-like gene product	-3.28	-6.53
Lj2g3v2830880	PREDICTED: abscisic acid receptor PYL4-like	-4.62	-7.52

Table S5. Twenty most up- and down-regulated *S. meliloti* genes after two and four days of water deprivation.

Gene ID	Description	log. FC	
		T2 vs. T0	T4 vs. T0
DU99_26890	antitoxin	2.91	1.38
DU99_30990	histidine kinase	2.43	-0.22
DU99_30995	histidine kinase	2.19	-0.55
DU99_27180	DNA topoisomerase	2.10	-0.64
DU99_30705	transcriptional regulator	1.94	-0.76
DU99_23220	uknown protein	1.87	-0.75
DU99_17140	uknown protein	1.68	-0.42
DU99_04335	radical SAM protein	1.59	-0.42
DU99_22915	uknown protein	1.58	-0.41
DU99_28870	uknown protein	1.52	0.46
DU99_07735	RelE toxin	1.30	-0.88
DU99_05870	MarR family transcriptional regulator	1.19	-0.86
DU99_14895	uknown protein	1.11	0.38
DU99_27175	glycogen debranching protein	1.10	-0.08
DU99_03830	uknown protein	1.06	0.91
DU99_09345	LysR family transcriptional regulator	1.05	-0.27
DU99_06725	DNA polymerase III subunit alpha	1.05	-0.08
DU99_03800	uknown protein	1.04	1.24
DU99_05145	cation\x3aproton antiporter	-1.29	-0.70
DU99_23265	Nodulation protein N	-2.65	-0.66
DU99_16195	homogentisate 1,2-dioxygenase	0.91	2.38
DU99_16200	MarR family transcriptional regulator	0.78	1.48
DU99_25085	uknown protein	0.81	1.38
DU99_25090	membrane protein	0.70	1.35
DU99_29415	guanine deaminase	-0.17	1.25
DU99_27195	inosine-5-monophosphate dehydrogenase	1.06	1.16
DU99_16190	2-keto-4-pentenoate hydratase	0.40	1.14
DU99_04230	photosystem reaction center subunit H	0.79	1.12
DU99_28340	uknown protein	0.80	1.07
DU99_16185	maleylacetoacetate isomerase	0.53	1.07
DU99_20315	MarR family transcriptional regulator	-0.40	-1.04
DU99_18380	penicillin-binding protein	-0.24	-1.17
DU99_00220	Phosphoenolpyruvate carboxykinase	-0.72	-1.20
DU99_00725	pilus assembly protein	-0.27	-1.35
DU99_12190	cell division protein FtsQ	-0.10	-1.38
DU99_32055	amino acid ABC transporter substrate-binding protein	-0.23	-1.48
DU99_09255	membrane protein	-0.66	-1.53
DU99_07710	nitrite reductase	-0.72	-1.61
DU99_26030	cell division protein FtsK	-0.23	-1.87
DU99_32940	MFS sugar transporter	-0.84	-3.01

Table S6. Twenty most up- and down-regulated *M. loti* genes after four days of water deprivation.

Gene ID	Description	log. FC	
		T2 vs. T0	T4 vs. T0
msr8579	uknown protein	2.80	6.19
msr9689	uknown protein	2.23	6.06
msl2054	uknown protein	2.51	5.99
mll1786	uknown protein	2.01	5.85
msl9563	uknown protein	2.59	5.82
msr7945	uknown protein	2.13	5.82
mll1958	uknown protein	2.45	5.81
mlr9657	uknown protein	2.56	5.81
msl7942	uknown protein	2.90	5.81
mlr0376	uknown protein	1.99	5.81
msl1750	uknown protein	2.09	5.78
msr8675	uknown protein	1.91	5.74
msr0042	uknown protein	2.11	5.73
msr8120	uknown protein	2.73	5.67
msr4805	uknown protein	1.81	5.62
msl9670	uknown protein	1.26	5.57
mll6859	uknown protein	2.06	5.56
msl3831	uknown protein	2.20	5.56
msl1572	uknown protein	1.90	5.55
msl7604	uknown protein	1.82	5.52
mlr2932	chemotaxis motc protein	-0.63	-2.72
mlr0308	50S ribosomal protein L5	-1.04	-2.72
mlr7768	porin	-0.71	-2.73
mlr3345	glutathione transferase	-0.75	-2.74
mlr2935	two-component response regulator	-0.78	-2.74
mlr2959	uknown protein	-0.52	-2.75
msl0722	uknown protein	-0.58	-2.76
mlr2912	flagellar basal-body rod protein\x3b FlgC	-1.12	-2.76
mlr2921	flagellar L-ring protein\x3b FlgH	-0.42	-2.84
mlr0480	uknown protein	-0.88	-2.86
mll6123	5-methyltetrahydropteroyltriglutamate-homocysteine methyltransferase	-0.52	-2.86
mlr5157	uknown protein	-0.76	-2.87
mlr3356	phosphonate ABC transporter (permease)	-0.68	-2.94
msr1274	sarcosine oxidase delta subunit	-0.35	-2.96
mlr2913	flagellar hook-basal body protein\x3b FleE	-1.42	-2.96
mll2946	uknown protein	-0.62	-2.99
mlr2911	flagellar basal-body rod protein\x3b FlgB	-0.96	-3.00
mlr2928	flagellar basal-body MS-ring protein\x3b FliF	-0.28	-3.03
mlr2924	flagellar biosynthesis protein\x3b FliP	0.06	-3.30
mll5088	S-adenosyl L-homocystein hydrolase	-0.67	-3.66

Table S7. Real-time qPCR conditions for plant genes expression analysis.

Temperature	Time
PCR	
50 °C	20 s
95 °C	10 min
40 cycles:	
95 °C	15 s
60 °C	1 min
Melting curve	
60 °C -> 95 °C (in 0.5 °C inc.)	5 s / step

Table S8. Real-time qPCR conditions for bacteria genes expression analysis.

Temperature	Time
PCR	
95 °C	2 min
40 cycles:	
95 °C	15 s
60 °C	30 s
Melting curve	
60 °C -> 95 °C (in 0.5 °C inc.)	5 s / step

Table S9. Accessions of genes, sequences of primers and product lengths for real-time qPCR. bp - base pairs.

Gene	Forward primer sequence 5′ → 3′	Reverse primer sequence 5′ → 3′	Product length [bp]
SmRpoE1 - RNA polymerase sigma factor protein	AGGAAGAGGTCCTGGAATTGAT	TCCTGGTAGCGATAACCCTCTA	367
SmNodC - NodC N-acetylglucosaminyltransferase	GGAGTATTGGCTTGCCTGTAAC	CCAGTCGCTATTGGACAATGTA	622
MlRecA - recombinase A	AGAGGATAAGGCAGTGGACAAA	GAGATCAGCAGGTTTTCAAGGT	357
MlNodC - chitooligosaccharide synthase NodC	CTAGCAATCGCAGTGAAACTTG	CCAATCGCTGTTGCTTAATGTA	665
MtFAR - F-box/ankyrin repeat SKIP35-like protein	CCATGTCCAGTTCTCTTCATTG	ATTGTCACTACTCCCAAGCTCAT	89
MtERD - early-responsive to dehydration stress	ACCACGTTTGTTAGCTGAAGG	TCCTCAGAAAGGTTCCAAGC	105
MtDEH - dehydrin	ACAAGCCATGGCAGATACAAC	ATCAACAAAAACACCGTCGTC	67
LjERD - early-responsive to dehydration stress	CATTCCTTCCCCTGATTGG	TGATAATGAGAGAAGTTGATCCTC A	67
LjDEH - dehydrin	AAAGGAACAAGATCATCAAAAACC	ACGTTGTCAAACTCAGTGAGGA	72
MtFAR - F-box/ankyrin repeat SKIP35-like protein	CCATGTCCAGTTCTCTTCATTG	ATTGTCACTACTCCCAAGCTCAT	89
MtPLT2 - PLT2 Integrase-type DNA-binding superfamily protein	CAAACGAAGTTCCAAAGGTTG	GGCTGCAAGATCTGACTCATT	77
MtAIL5 - AIL5 AINTEGUMENTA-like 5	TCTTTTCCACTCCTTCACACC	GGGTTGTTGCTTCCTGTGAG	75
MtAIL6 - AIL6 AINTEGUMENTA-like 5	GAACAATCAATCTGGTTTGTGG	TGTGTTTCTGTTTGACTGTCACC	98
MtANT_1 - ANT Integrase-type DNA-binding superfamily protein 1	TTGACATTAACAATGAAAATAACTGG T	TGATGAGTGTGTGAAGAAACACC	85
MtANT_2 - ANT Integrase-type DNA-binding superfamily protein 2	TCTCAACATCATCATCCTAGTCATC	TGACATCAAGTAAAAGTTAGAGG GAAC	96
MtANT_3 - ANT Integrase-type DNA-binding superfamily protein 3	GTTTCTCTCTCTCACCCCACA	GAAGTTTGAGTATGTTGGTAATGA TGA	70
MtBBM - BBM Integrase-type DNA-binding superfamily protein	CTCATCAGCCGGAAGCAT	GTTTCTGAGCCATGTTTTTATCATA G	95
MtCCD7 - CCD7 carotenoid cleavage dioxygenase 7	CCCAACCCCTCATCTTACAA	TTGGTTATTGTTTGGGGTAAGG	83

MtCCD8 - CCD8 carotenoid cleavage dioxygenase 8	CGGAGAAACGTTCAGAGACC	TTCCCATCTCTCTTGATGGATAC	77
MtSDR2 - SDR2 NAD(P)-binding Rossmann-fold superfamily protein	TCTCTTCACTCTACAGACACCACA	ATTTTTCCTTCCAACCTCTTAGG	62
MtNCED4 - NCED4 nine-cis-epoxycarotenoid dioxygenase 4	TGGTCCCTAAACCCATCATAA	TGGTTTTGATGGATGAGATGTT	87
MtNCED5 - NCED5 nine-cis-epoxycarotenoid dioxygenase 5	CAACACAAATGGAACCTTCTACA	GATTAAGGTTGTTTCGACGAAGTC	69
LjPP2AA - protein phosphatase 2A regulatory subunit A	TGAGCTATGTGAAGCTGTTGGT	CAGCCTCATTATCACGCAGTAG	93
LjPLT2 - PLT2 Integrase-type DNA-binding superfamily proteinMtNCED4 - NCED4 nine-cis-epoxycarotenoid dioxygenase 4	CGTGGAGTTACAAGGCATCATGGTCC CTAAACCCATCATAA	CTGCAACTCTTCCAATCCTTGTGG TTTTGATGGATGAGATGTT	61
LjAIL5 - AIL5 AINTEGUMENTA-like 5MtNCED5 - NCED5 nine-cis-epoxycarotenoid dioxygenase 5	GCTTATGATCTTGCAGCTCTCACAAC ACAAATGGAACCTTCTACA	GTTGCAAATTGGGAAGTTGGGATT AAGGTTGTTTCGACGAAGTC	66
LjAIL6 - AIL6 AINTEGUMENTA-like 6LjPP2AA - protein phosphatase 2A regulatory subunit A	CTACGGCGAGGAGCAAGTTGAGCTAT GTGAAGCTGTTGGT	CAACGCGTTCATGAAAAGAACAG CCTCATTATCACGCAGTAG	64
LjANT- ANT Integrase-type DNA-binding superfamily proteinLjSCR_2 - SCR GRAS family transcription factor 2	GCTGAAGTGGTTCCACCAAGATCATG ATTCGTCCCCACA	AGTCCATAATAGAAGCCATAGTT GCAGTGATGATCAGCAATGTTTGG	71
LjCCD7 - CCD7 carotenoid cleavage dioxygenase 7LjPLT2 - PLT2 Integrase-type DNA-binding superfamily protein	CAACATACATTCCTCCCCTCATCGTG GAGTTACAAGGCATCA	TTGGTTACCAGCGGCAAGCTGCA ACTCTTCCAATCCTTG	67
LjCCD8 - CCD8 carotenoid cleavage dioxygenase 8LjAIL5 - AIL5 AINTEGUMENTA-like 5	CAAATGTTGCAAGTCCATCAGGCTTA TGATCTTGCAGCTCTCA	TCATCGGAGGTCCCTGACGTTGCA AATTGGGAAGTTGG	70
LjSDR2 - SDR2 NAD(P)-binding Rossmann-fold superfamily proteinLjAIL6 - AIL6 AINTEGUMENTA-like 6	GGAAGGTAAAGTCGCCATTGCTACGG CGAGGAGCAAGT	AAAACTCTCACCGTTGCTTCACAA CGCGTTCATGAAAAGAA	66
LjNCED4 - NCED4 nine-cis-epoxycarotenoid dioxygenase 4LjANT- ANT Integrase-type DNA-binding superfamily protein	CACGCCCTCCGTATCTCAGCTGAAGT GGTTCCACCAAG	TTTTGTATTTGTATGTTTTGACGTA GCAGTCCATAATAGAAGCCATAG TTGC	70
LjNCED5 - NCED5 nine-cis-epoxycarotenoid dioxygenase 5LjCCD7 - CCD7 carotenoid cleavage dioxygenase 7	CATTCTATGACTTGGGCTTTCCCAACA TACATTCCTCCCCTCAT	TGTAGAGCACAATCCACCTTTCTT GGTTACCAGCGGCAAG	61
Ml16SrRNA - 16S ribosomal RNALjCCD8 - CCD8 carotenoid cleavage dioxygenase 8	GCCTTTGATACTGGGTATCTCGCAAAT GTTGCAAGTCCATCAG	GAATTTCACCTCTACACTCGGAAT TCATCGGAGGTCCCTGAC	67

MlKu - Ku protein LjSDR2 - SDR2 NAD(P)-binding Rossmann-fold superfamily protein	CTATGTCGATGCCGAAACCGGAAGGTAAAGTCGCCATTG	TCCAATTCATAGCCCTTGATCTAAAACTCTCACCGTTGCTTCA	66
MlHisKin_1 - sensory transduction DNA-binding kinaseLjNCED4 - NCED4 nine-cis-epoxycarotenoid dioxygenase 4	GCCTATCCCGAGGTCGATACCACGCCCTCCGTATCTCA	CAAGGTCGAAAGGCTTGGTTTTGTATTTGTATGTTTTGACGTAGC	85
MlHisKin_3 - sensory transduction DNA-binding kinaseLjNCED5 - NCED5 nine-cis-epoxycarotenoid dioxygenase 5	TCAGGTTTGGGTCGATGCCATTCTATGACTTGGGCTTTCC	GCTTCTGCTCGGTCGTCTTGTAGAGCACAATCCACCTTTC	86
MlOtsA - OtsA trehalose-6-phosphate synthase Ml16SrRNA - 16S ribosomal RNA	AACGCTGCGTCACATCCTGCCTTTGATACTGGGTATCTCG	CACGGTTTGAGACGACGATGAATTTCACCTCTACACTCGGAAT	65
MlCarS - cardiolipin synthase phospholipase D family protein MlKu - Ku protein	TCGACCGATAAAATCAAAGCACTATGTCGATGCCGAAACC	GCGAACGCATCGTAATTGTTCCAATTCATAGCCCTTGATCT	68
MlHisKin_1 - sensory transduction DNA-binding kinase	GCCTATCCCGAGGTCGATAC	CAAGGTCGAAAGGCTTGG	85
MlHisKin_3 - sensory transduction DNA-binding kinase	TCAGGTTTGGGTCGATGC	GCTTCTGCTCGGTCGTCT	86
MlOtsA - OtsA trehalose-6-phosphate synthase	AACGCTGCGTCACATCCT	CACGGTTTGAGACGACGAT	65
MlCarS - cardiolipin synthase phospholipase D family protein	TCGACCGATAAAATCAAAGCA	GCGAACGCATCGTAATTGT	68

Table S10. The confirmation of RNA-Seq results by real-time qPCR.

Protein / Gene ID		RNA-Seq results			Real-time qPCR results		
		**T0vsR_FC**	**T2vsT0_FC**	**T4vsT0_FC**	**T0vsR_FC**	**T2vsT0_FC**	**T4vsT0_FC**
**PLT2 Integrase-type DNA-binding superfamily protein**							
MTR_2g098180	*MtPLT2*	1.21	-1.13	-1.32	-2.68	2.14	1.61
Lj3g3v3245260	*LjPLT2*	-33.41	-1.59	-1.59	-213.74	-1.06	-1.52
**AIL5 AINTEGUMENTA-like 5**							
MTR_4g127930	*MtAIL5*	-2.22	-1.17	-1.58	-3.50	-1.35	-1.40
Lj4g3v2120330	*LjAIL5*	-2.20	1.39	2.27	-2.89	1.25	2.53
**AIL6 AINTEGUMENTA-like 6**							
MTR_5g031880	*MtAIL6*	5.69	-1.42	-2.11	2.67	-1.17	-1.12
Lj2g3v1455120	*LjAIL6*	-7.18	-1.40	-1.98	-9.86	-1.63	-2.09
**ANT Integrase-type DNA-binding superfamily protein**							
MTR_4g097520	*MtANT_1*	-9.54	1.03	1.01	-23.52	1.14	1.25
MTR_1g017400	*MtANT_2*	-8.73	1.17	1.21	-23.68	1.29	1.35
MTR_3g103460	*MtANT_3*	-7.15	1.19	-1.16	-10.77	1.47	1.25
Lj4g3v0708640	*LjANT*	-17.71	1.28	-1.22	-21.81	1.41	-1.44
**BBM Integrase-type DNA-binding superfamily protein**							
MTR_7g080460	MtBBM	3.35	-1.27	-2.19	2.18	-1.09	-1.54
**NCED4 nine-cis-epoxycarotenoid dioxygenase 4**							
MTR_5g025270	*MtNCED4*	-1.01	-1.06	1.13	1.04	-1.68	-1.51
Lj2g3v1561960	*LjNCED4*	-6.32	2.86	2.19	-9.15	1.95	1.73
**NCED5 nine-cis-epoxycarotenoid dioxygenase 5**							
MTR_2g070460	*MtNCED5*	3.15	1.07	-1.48	6.27	-2.93	-4.21
Lj4g3v2467280	*LjNCED5*	-2.90	-1.11	1.19	-3.47	1.28	1.66
**SDR2 NAD(P)-binding Rossmann-fold superfamily protein**							
MTR_7g071680	*MtSDR2*	-1.05	-3.88	-3.51	-1.78	-3.14	-1.80
Lj0g3v0160209	*LjSDR2*	-2.69	-4.17	-8.17	-4.25	-5.76	-10.24
**Ku protein**							
mll4607	*MlKu*	-	2.28	14.17	-	3.75	36.42
**Sensory histidine kinase 1**							
mll1133	*MlHisKin_1*	-	1.67	1.86	-	1.05	1.39
**Sensory histidine kinase 3**							
mlr3704	*MlHisKin_3*	-	3.65	19.47	-	4.91	27.32
**OtsA trehalose-6-phosphate synthase**							
mll0691	*MlOtsA*	-	4.47	17.55	-	3.65	15.69
**Cardiolipin synthase**							
mlr9675	*MlCarS*	-	2.67	17.59	-	2.45	15.72
